# PGK1 Regulates Oxidative Stress in Gestational Diabetes Mellitus through the Estradiol-Keap1-Nrf2 Pathway

**DOI:** 10.7150/ijbs.113728

**Published:** 2025-08-25

**Authors:** You Peng, Hengli Zhao, Jun Chen, Chi Chiu Wang, Tao Zhang, Tsz Ching Yeung, Haotong Ouyang, Jiayu Zhu, Xiangli Chen, Meng Li, Haoyue Hu, Mei Zhong

**Affiliations:** 1 Department of Obstetrics and Gynecology, Nanfang Hospital, Southern Medical University, Guangzhou, 510515, China.; 2 Guangdong Provincial Key Laboratory of Clinical Pharmacology, Medical Research Institute, Guangdong Provincial People's Hospital, Guangdong Academy of Medical Sciences, Southern Medical University, Guangzhou 510080, China.; 3 Department of Obstetrics and Gynecology, Li Ka Shing Institute of Health Sciences; School of Biomedical Sciences; and The Chinese University of Hong Kong-Sichuan University Joint Laboratory in Reproductive Medicine, The Chinese University of Hong Kong, Hong Kong, China.; 4 Department of Obstetrics and Gynecology, Faculty of Medicine, Prince of Wales Hospital, The Chinese University of Hong Kong, Hong Kong, China; 5 Department of Epidemiology, School of Public Health, Southern Medical University, Guangzhou, 510515, Guangdong, China.; 6 Department of Obstetrics, The Seventh Affiliated Hospital, Southern Medical University, Foshan, 528244, China.; 7 Reproductive Medicine Center, The Eighth Affiliated Hospital of Southern Medical University (The First People's Hospital of Shunde), Foshan, 528300, Guangdong, China.; 8 Sino-US Center of Translational Medicine for Development Disabilities, Southern Medical University, Guangzhou, Guangdong, China.

**Keywords:** Gestational diabetes mellitus, PGK1, Estradiol, Keap1-Nrf2-ARE signaling, Keap1 dimerization

## Abstract

Gestational diabetes mellitus (GDM), the most common pregnancy-related metabolic disorder, is characterized by exacerbated oxidative stress (OS). The inhibition of phosphoglycerate kinase 1 (PGK1), the first ATP-generating enzyme in the glycolytic pathway, activates Keap1-Nrf2 antioxidant pathways and reduces OS. However, the detailed roles of PGK1 in GDM remain unexplored. Disruption of pro-oxidant/antioxidant homeostasis was observed in the placentas of GDM patients. PGK1 was significantly upregulated in both human GDM placentas and streptozotocin (STZ)-induced model mice. Pharmacological inhibition of PGK1 *in vivo* ameliorated placental dysfunction, attenuated excessive ROS production, and improved pregnancy outcomes. Lentivirus-mediated PGK1 knockdown in HTR8/SVneo trophoblasts increased Nrf2-dependent antioxidant protein expression while reducing ROS generation. Mechanistically, PGK1 inhibition elevated estradiol levels, facilitating Keap1 dimerization, and this dimerization destabilized the Keap1-Nrf2 complex, enabling Nrf2 accumulation and antioxidant activation. Exogenous estradiol supplementation recapitulated the effect of inhibiting PGK1 by enhancing Keap1 dimer formation, effectively mitigating placental OS and adverse pregnancy phenotypes in GDM models. This study elucidates the critical role of PGK1 in restoring redox homeostasis through the estradiol-Keap1-Nrf2 axis in the pathogenesis of GDM. PGK1/estradiol crosstalk represents a druggable target, and pharmacological PGK1 inhibition has translational potential for mitigating oxidative stress-related pregnancy complications.

## Introduction

Gestational diabetes mellitus (GDM) is characterized by metabolic disturbance during pregnancy and affects approximately 14.0% of pregnancies worldwide 1,2. It is defined as hyperglycemia due to the failure of pancreatic β-cells to match the increased insulin requirements during pregnancy 1. High-glucose (HG) environments can activate the glycolysis pathway and induce excessive reactive oxygen species (ROS) production, increasing oxidative stress (OS), which causes mitochondrial defects, cellular apoptosis, and inflammation 3,4. This process has been proposed to be a hallmark of the early pathogenesis of GDM 5. Moreover, a growing number of studies have demonstrated that certain enzymes of the glycolytic pathway may act as regulatory targets of OS 6,7. Therefore, inhibiting excessive ROS generation and suppressing OS may be therapeutic strategies for ameliorating GDM.

Phosphoglycerate kinase 1 (PGK1), the first ATP-generating enzyme in the glycolytic pathway, catalyzes the generation of 3-phosphoglycerate (3-PG) and ATP. Owing to its rate-limiting role in regulating ATP and 3-PG levels, PGK1 influences energy production and redox balance 8. PGK1 can regulate OS by regulating the Kelch-1-like ECH-associated protein l (Keap1)/nuclear factor erythroid 2-related factor 2 (Nrf2) pathway 9-11. Nrf2, a major transcription factor, regulates cellular antioxidant defense pathways by activating antioxidant response element (ARE)-responsive downstream genes, including NAD(P)H-quinone oxidoreductase 1 (NQO1) and heme oxygenase-1 (Ho-1) 11,12. Under normal conditions, Nrf2 is constitutively ubiquitinated by Keap1 through the Nrf2-ECH homology domain (Neh2 structural domain) and Cullin-3 E3 ligase and is subsequently degraded by the proteasome 13. In response to OS, the Keap1-Nrf2 complex dissociates, which results in Nrf2 translocation into the nucleus, where it initiates the transcription of antioxidant genes at ARE loci 11,12. Moreover, recent studies have reported that inhibiting PGK1 can increase the dimerization of Keap1 and increase Nrf2 accumulation, which activates the Nrf2 signaling pathway 11. However, the function of PGK1 in GDM and the direct link between PGK1 and Nrf2 remain to be investigated.

Currently, lifestyle interventions, including dietary modification, exercise training, and weight management, have shown beneficial effects for GDM; however, this strategy fails to sustain blood glucose control in 15-30% of patients with GDM 14. Therefore, effective medical treatments for GDM are still lacking. This study aimed to explore the interaction between glucose metabolism and placental OS and identify new targets for GDM therapy by restoration of metabolic homeostasis.

Here, we report an imbalance in pro-oxidant/antioxidant homeostasis in the placentas of women with GDM and that increased glycolysis induced by high-glucose environments causes placental OS. To determine the role of PGK1, we first investigated the expression of PGK1 in the placentas of GDM patients and model mice and confirmed the beneficial effect of PGK1 inhibition on OS. Mechanistically, we found that PGK1 activates the Nrf2-ARE pathway by regulating the dimerization of Keap1; Keap1 dimerization promotes Keap1-Nrf2 complex dissociation and increases Nrf2 accumulation. Furthermore, decreased PGK1 leads to the accumulation of the reactive metabolite estradiol. Exogenous estradiol supplementation recapitulated these effects in a receptor-independent manner by enhancing Keap1 dimer formation, ultimately reducing placental OS and rescuing adverse pregnancy phenotypes in GDM models. These results establish PGK1-driven metabolic reprogramming via the estradiol-Keap1-Nrf2 axis as a critical pathway in GDM pathogenesis, shed new light on the metabolic regulation of OS in GDM, and provide a novel therapeutic strategy for GDM.

## Materials and Methods

### Patients and placenta collection

From 2020 to 2022, a total of 16 normal pregnant women (NCs) and 16 patients with GDM were recruited at the Department of Obstetrics and Gynecology, Southern Medical University Nanfang Hospital, China. The diagnostic criteria for GDM were based on those recommended by the International Association of Diabetes and Pregnancy Study Groups (IADPSG): fasting plasma glucose ≥ 5.1 mmol/L and/or 1-h PG ≥ 10.0 mmol/L and/or 2-h PG ≥ 8.5 mmol/L. GDM patients with other pregnancy complications and a history of diabetes mellitus were excluded. The NC group included pregnant women without any pregnancy complications or any underlying disease.

After delivery, six 1 cm^3^ portions of placental tissue were cut from the fresh placentas around the umbilical cords, covering both the maternal and fetal sides. After being washed in a normal saline solution several times, one portion was fixed in 4% paraformaldehyde for histological and immunological analysis, and the other portions were cut completely after removing blood vessels, amniotic membranes and maternal decidua and stored at -80 °C before use. Clinical characteristics were obtained from standardized medical records and are presented in Table 1.

### Animal study

Eight-week-old C57BL/6J mice, purchased from the Guangdong Medical Laboratory Animal Center, were housed in a pathogen-free environment with a 12-hour light/dark cycle at 23 ± 3 °C. The female mice were randomized into four groups: the NC group, the GDM group, the GDM+NG52 group, and the GDM+estradiol group. Except for the NC group, which was given a normal diet throughout pregnancy, the other three groups were comprised of GDM model animals and were fed a high-fat diet (HFD; 60% energy from fat; GDMLAC, China) for 4 weeks before pregnancy.

The mice were mated at a 1:2 ratio of males to females overnight and the female mice were monitored for vaginal plug formation. If a vaginal plug formed, it was identified as gestational day (GD) 0.5. On GD 6.5, STZ (30 mg/kg; Solarbio, China) was intraperitoneally injected into the GDM group three times every 8 hours for a total of 3 times, and the same amount of citrate buffer was injected into the NC group (n = 15). Random blood glucose was tested from the tail by a glucometer (Sinocare, China) on GD 9.5. GDM mice with random blood glucose levels ≥11.1 mmol/L were divided into three groups. The GDM group (n = 15) was administered vehicle, the NG52 group (n = 5) was administered NG52 (100 mg/kg; MCE, USA) dissolved in 0.5% CMC-Na (Solarbio, China) by gavage, and the estradiol group (n = 5) was intraperitoneally injected with estradiol solution (0.2 mg/kg/day; MCE, USA) dissolved in olive oil from GD 9.5 to GD 18.5.

On GD 18.5, blood glucose levels were detected and recorded, after which the mice were anesthetized and sacrificed. The placentas and fetuses were separated from the uterus, weighed, and recorded. Serum samples were collected for ELISA. Mouse placentas were stored at -80 °C or fixed in 4% paraformaldehyde for later analysis.

To investigate the dosage effects of estradiol, we established a virgin mouse group (VM, n = 5) using 8-week-old female C57BL/6J mice. Whole blood was collected from non-estrus phase animals via retro-orbital bleeding, and serum was isolated for ELISA detection.

### Cell culture and treatment

HTR8/SVneo cells were purchased from the American Type Culture Collection (ATCC, USA) and cultured under standard conditions in RPMI-1640 medium (Gibco, USA) supplemented with 12% fetal bovine serum and 1% penicillin‒streptomycin.

HTR8/SVneo cells were seeded, cultured to 70% confluence, and then treated with 25 mM glucose (HG) or 5.5 mM glucose (NC) for 24 h. CBR-470-1 (MCE, USA) was diluted to 20 μM with HG medium for cell stimulation.

For the knockdown experiments, a set of shRNA oligonucleotides (Table 2) against human PGK1, Keap1, and Nrf2 were individually annealed, subcloned, and inserted into the pLKO.1-puro vector by Tsingke (Beijing, China). For PGK1 overexpression, full-length PGK1 was synthesized, cloned, and inserted into the TK-PCDH-copGFP-T2A-Puro vector by Tsingke (Beijing, China). Lentivirus packaging and infection were performed as previously described 15. The lentivirus was added directly to HTR8/SVneo cells at 40% confluence in complete RPMI-1640 medium supplemented with 10 µg/mL polybrene. After selection with 2.0 μg/mL puromycin (MCE, USA), stable cell lines were established and verified by quantitative real-time PCR (RT‒qPCR) and Western blot.

To construct a stable cell strain in which PGK1 was concomitantly knocked down with Keap1 or Nrf2, two lentiviruses were used to infect HTR8/SVneo cells simultaneously, and the subsequent steps were the same.

When investigating the role of ER receptors, we first treated cells with HG for 12 hours, followed by stimulation with 0.5 μM AZD9496 or 0.5 μM Fulvestrant for an additional 12 hours. Subsequently, 100 nM estradiol was added to each well and incubated for 24 hours before protein extraction for analysis.

### Statistical analysis

All experiments had at least 3 replicates. The results are presented as the means ± SDs. All the statistical analyses were performed using GraphPad Prism 8.0 or SPSS 19.0 software. A normality test was conducted to determine whether to use a parametric or nonparametric test. Significant differences between two or more independent groups were determined by two-tailed unpaired Student's t test and one-way ANOVA, respectively, followed by Tukey's post hoc test. Statistical significance was defined as a* P* value ≤ 0.05.

The detailed methods are available in the Data Supplement.

## Results

### OS is activated in GDM human and mouse model placentas

**Figure 1:**
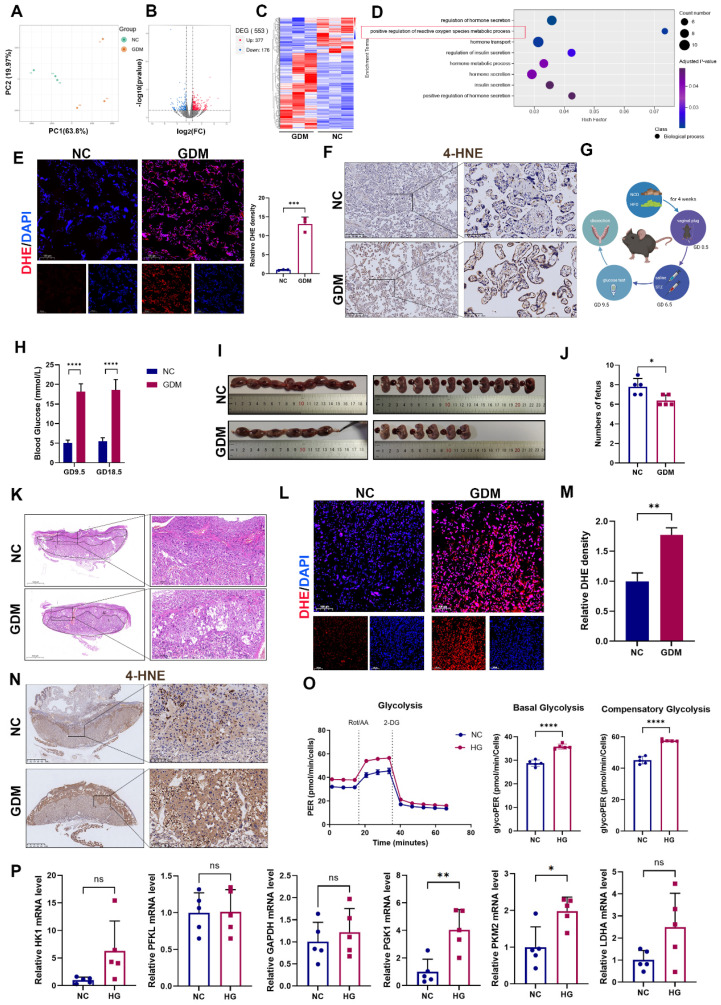
** Oxidative stress (OS) is increased in both human and mouse GDM placentas.** (A) Transcriptomic principal component analysis (PCA) of placental tissues from GDM patients versus normal controls (NC); n = 3. (B) Volcano plot of differentially expressed genes (DEGs) between NC and GDM human placentas. (C) Heatmap of expression patterns in NC and GDM human placentas. (D) Gene Ontology enrichment analysis of upregulated DEGs in GDM human placentas. (E) Representative immunofluorescence (IF) images and quantitative analysis of ROS (red) in human placental tissues. Nuclei were stained with DAPI (blue); scale bar = 100 μm; n = 3. (F) Representative immunohistochemical (IHC) staining of 4-hydroxynonenal (4-HNE) in NC and GDM human placentas. Left: Overview (scale bar = 500 μm). Right: High magnification (scale bar = 100 μm). (G) Schematic diagram of the mouse treatments. (H) Blood glucose levels of the mice; n = 5. J) Representative images (I) and fetal quantification (J) in NC and GDM mice; n = 5. H&E staining of mouse placental sections. Left: Overview (scale bar = 1,000 μm). Right: High magnification (scale bar = 200 μm). M) Representative IF images (L) and quantitative analysis (M) of ROS (red) in mouse placental tissues. Nuclei were stained with DAPI (blue); scale bar = 100 μm; n = 3. Representative IHC staining of 4-HNE in NC and GDM mouse placentas. Left: Overview (scale bar = 1 mm). Right: High magnification (scale bar = 100 μm). (N) Extracellular acidification rate (ECAR) measurement. Treatments of cells with rotenone/antimycin A (Rot/AA; 0.5 μM) and 2-deoxyglucose (50 mM) are indicated. Basal glycolysis was measured before Rot/AA injection. The compensatory glycolysis rate was the maximum glycoPER after Rot/AA injection; n = 5. (O) Quantitative real-time PCR (RT‒qPCR) analysis of HK1, PFKL, GAPDH, PGK1, PKM2, and LDHA in HTR8/SVneo cells stimulated with high glucose (HG). Relative mRNA levels were calculated and normalized to the β-actin level using the 2^-△△Ct^ method; n = 5. * represents* p* < 0.05, ** represents* p* < 0.01, *** represents* p* < 0.001, **** represents* p* < 0.0001 and ns represents *p* > 0.05.

To investigate the distinct molecular mechanisms underlying NC and GDM pregnancies, we conducted RNA sequencing analysis of placental tissues obtained from NC and GDM patients. Principal component analysis (PCA) revealed a clear separation between the placental transcriptomic profiles of the GDM and NC groups (Fig. 1A). Comparative analysis revealed 377 significantly upregulated and 178 significantly downregulated genes in GDM placentas compared with NC controls (Fig. 1B and 1C). Previous studies have reported that OS is critical in the pathogenesis of GDM 16. GO enrichment analysis also revealed that the regulation of ROS was downregulated in the placentas of women with GDM (Fig. 1D). This finding was further validated by placental dihydroethidium (DHE) staining, which revealed markedly elevated ROS levels in the GDM group compared with the control group (Fig. 1E). The levels of 4-Hydroxynonenal (4-HNE), a major lipid peroxidation product that appears during OS 17, were higher in the placentas of GDM patients than in those of the NC group (Fig. 1F).

Although increased OS in GDM placentas has been previously reported 16 and corroborated in our human placental samples, we further validated this phenomenon *in vivo* by establishing a murine GDM model (Fig. 1G). Blood glucose was detected three days after injection with STZ on GD9.5 and detected before anesthetization on GD18.5 (Fig. 1H). Assessment of pregnancy outcomes revealed a significant reduction in fetal number in the GDM group compared with the control group (Fig. 1I and 1J). Structurally, the mouse placenta is composed of 3 layers, including the maternal decidua, the middle junctional zone, and the vessel-dense labyrinth layer on the fetal side. Notably, the junctional zone serves as a critical nutrient reservoir for fetal development among these layers 18. Histological analysis demonstrated that in the GDM group, the middle junctional zone of the placenta had larger necrotic and infarction areas (Fig. 1K), which may have contributed to the observed adverse pregnancy outcomes. Moreover, both DHE staining and 4-HNE revealed that ROS levels were increased in the GDM group (Fig. 1L-1N).

Building upon established evidence that hyperglycemia induces glycolytic pathway activation 3, we first verified this metabolic shift using Seahorse Extracellular Flux analysis in HTR-8/SVneo cells, cultured under HG (25mM) conditions to mimic the hyperglycemic environment. Our results confirmed a significant increase in glycolytic flux under HG stimulation (Fig. 1O). Considering the multienzymatic complexity of the glycolytic cascade, we systematically screened glycolytic enzymes by RT‒qPCR and identified PGK1 as the most prominently activated enzyme under HG conditions (Fig. 1P).

Collectively, our integrated studies demonstrate that: in human and murine GDM models, HG-induced OS leads to placental dysfunction and fetal developmental compromise; at the cellular level, HG exposure triggers metabolic reprogramming characterized by enhanced glycolytic flux and PGK1 upregulation in trophoblasts.

### PGK1 levels are elevated and Keap1-Nrf2-ARE signaling is suppressed in human and mouse GDM placentas

**Figure 2:**
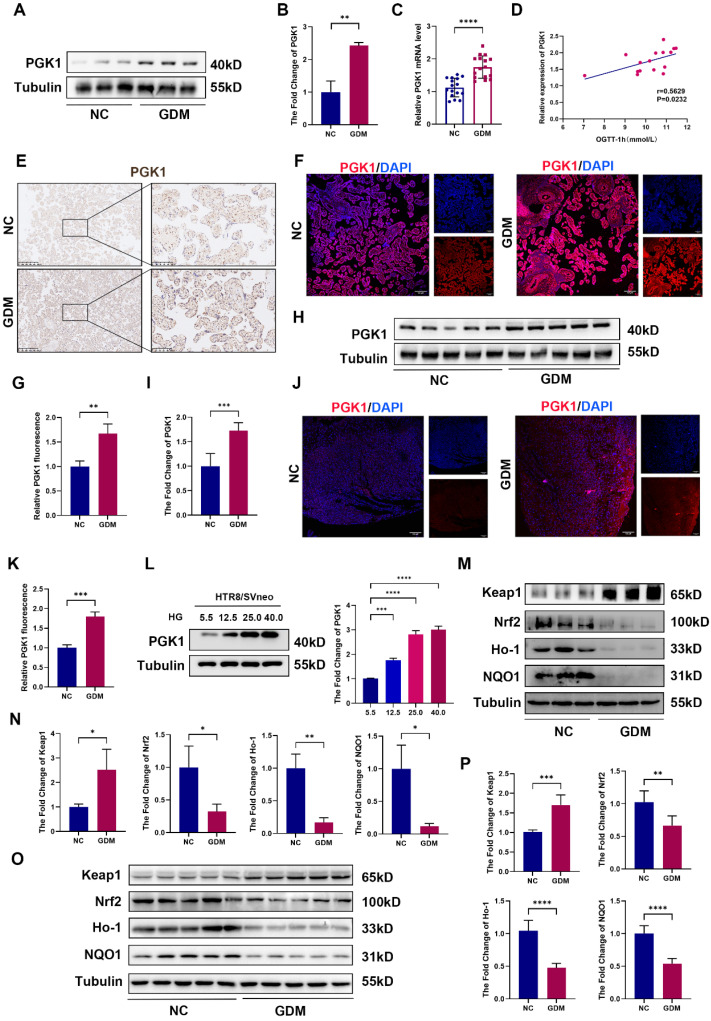
** PGK1 levels are elevated and Keap1-Nrf2-ARE signaling is suppressed in human and mouse GDM placentas.** Representative Western blot analysis of PGK1 expression in NC and GDM human placentas. (B) Quantitative analysis of PGK1 protein levels; n = 3. (C) RT‒qPCR analysis of PGK1 in human placental tissue. Relative mRNA levels were calculated and normalized to the β-actin level using the 2^-△△Ct^ method; n = 16. (D) Spearman correlation analysis between the oral glucose tolerance test (OGTT)-1 h values and placental PGK1 expression levels. (E) IHC analysis of PGK1 expression in human placental tissues. Left: Overview (scale bar = 500 μm). Right: High magnification (scale bar = 100 μm). (F-G) Representative IF images (F) and quantification (G) of PGK1 (red) in human placental tissues. Nuclei were stained with DAPI (blue); scale bar = 100 μm; n = 3. (H) Representative Western blot analysis of PGK1 expression in NC and GDM mouse placentas. (I) Quantitative analysis of PGK1 protein levels; n = 5. (J-K) Representative IF images (J) and quantification (K) of PGK1 (red) in mouse placental tissues. Nuclei were counterstained with DAPI (blue); scale bar = 100 μm; n = 3. (L) Representative Western blot images and quantification of the levels of PGK1 in HTR8/SVneo cells treated with different concentrations of glucose; n = 3. (M) Representative Western blot analysis of Keap1, Nrf2, Ho-1, and NQO1 in NC and GDM human placentas. (N) Quantitative analysis of Keap1, Nrf2, Ho-1 and NQO1 protein levels; n = 3. (O-P) Representative Western blot images (O) and quantification (P) of the levels of Keap1, Nrf2, Ho-1, and NQO1 in NC and GDM mouse placentas; n = 5. * represents *p* < 0.05, ** represents *p* < 0.01, *** represents *p* < 0.001 and **** represents *p* < 0.0001.

Given that PGK1 is the most significantly upregulated enzyme under HG stimulation and is known to play a pivotal role in metabolic disorders, we assessed its expression in placental chorionic villi tissues from NC and GDM patients. Compared with those in control placentas, the PGK1 levels in GDM placentas were markedly greater (Fig. 2A-2C). Moreover, PGK1 levels were positively correlated with blood glucose levels in GDM patients (Fig. 2D). The results of IHC and immunofluorescence further confirmed elevated PGK1 expression in GDM placentas (Fig. 2E-2G). Consistent with the human data, placental tissues from GDM model mice also exhibited significantly higher PGK1 protein levels than those from normal controls (Fig. 2H-2K). To validate these observations *in vitro*, we simulated HTR8/SVneo cells with different concentrations of glucose and observed a gradient-dependent increase in PGK1 levels within the concentration range of 5.5 mM to 25.0 mM (Fig. 2L).

Previous studies have demonstrated that inhibiting PGK1 increases the dimerization of Keap1 and promotes Nrf2 accumulation, which activates the Nrf2 signaling pathway. Considering that the Keap1‒Nrf2 pathway plays a vital role in regulating OS in GDM 19, we examined the expression of Keap1, Nrf2, and antioxidant proteins (Ho-1 and NQO1) in placental tissues. Our analysis revealed significantly elevated Keap1 protein levels accompanied by reduced expression of Nrf2, Ho-1, and NQO1 in the placentas of GDM patients compared with those of controls (Fig. 2M and 2N). Placental samples from GDM mice also presented increased Keap1 expression and decreased levels of Nrf2, Ho-1, and NQO1 (Fig. 2O and 2P).

Our multilevel investigation demonstrated that PGK1 induction in GDM placentas parallels antioxidant dysfunction, linking hyperglycemia to OS through Keap1-Nrf2 suppression.

### PGK1 inhibition improves pregnancy outcomes *in vivo* and alleviates trophoblast OS *in vitro*

**Figure 3:**
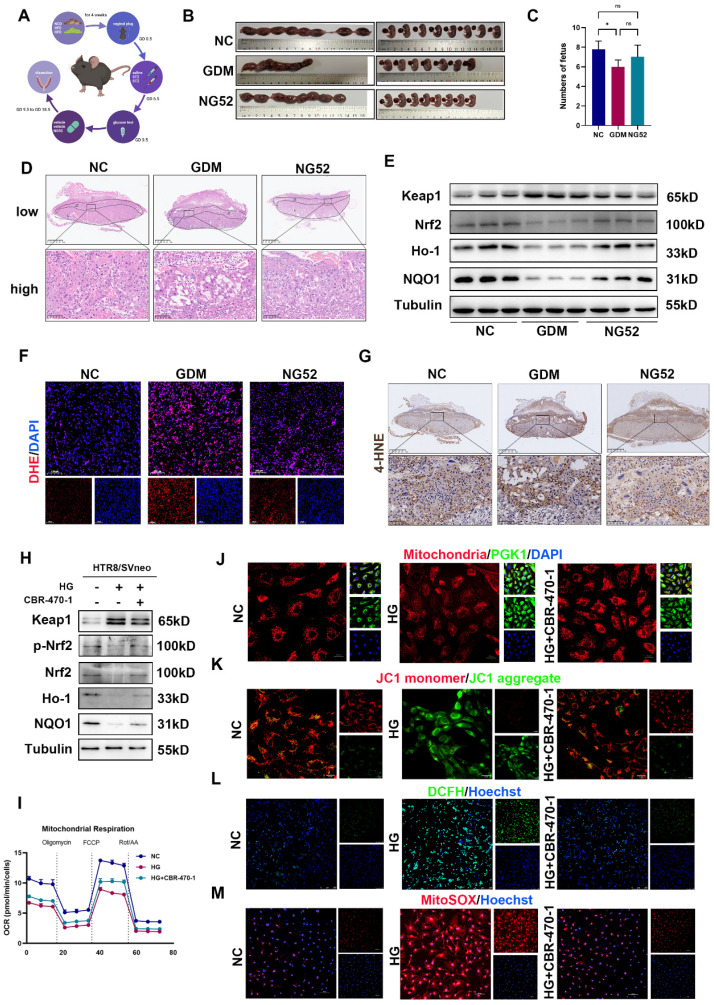
** PGK1 inhibition improves pregnancy outcomes *in vivo* and alleviates trophoblast OS* in vitro*.** Schematic diagram of mouse treatments. C) Representative images (B) and fetal quantification (C) of the control group, GDM group, and NG52 group; n=5. (D) H&E staining of mouse placental sections. Up: Overview (scale bar = 1,000 μm). Down: High magnification (scale bar = 100 μm); n = 5. (E) Representative Western blot images of Keap1, Nrf2, Ho-1, and NQO1 in the three groups. (F) IF analysis of ROS levels (red) in mouse placental tissues, and nuclei were counterstained with DAPI (blue); scale bar = 100 μm. Representative IHC staining of 4-HNE in NC, GDM, and NG52-treated mouse placentas. Up: Overview (scale bar = 1 mm). Down: High magnification (scale bar = 100 μm). Representative Western blot images of Keap1, p-Nrf2, Nrf2, Ho-1, and NQO1 levels in HTR8/SVneo cells. OCR measurements. The cells were treated with oligomycin (1.5 μM), FCCP (1 μM) or Rot/AA (0.5 μM) as indicated. The morphology of mitochondria were detected by staining with MitoSox™ red; scale bar = 20 μm. Representative fluorescence images showing MCMP in the various groups; scale bar = 20 μm. Representative fluorescence images showing DCFH-DA (green) staining in HTR8/SVneo cells, and nuclei were counterstained with Hoechst (blue); scale bar = 250 μm. Representative fluorescence images showing MitoSOX staining (red) of HTR8/SVneo cells and nuclei counterstained with Hoechst (blue); scale bar = 100 μm. * represents *p* < 0.05, ** represents *p* < 0.01 and ns represents *p* > 0.05.

To further clarify whether inhibiting PGK1 can improve pregnancy outcomes *in vivo*, we administered the PGK1 inhibitor NG52 to a GDM mouse model (Fig. 3A). The blood glucose levels in mice at GD18.5 were slightly lower after NG52 injection compared to the GDM group, but this difference was not statistically significant (Fig. S1A). The results revealed significantly poorer pregnancy outcomes in the GDM group, which were effectively ameliorated following NG52 treatment (Fig. 3B and 3C). H&E staining of placentas revealed that the necrotic and infarctional areas were decreased in the NG52 group compared with those in the GDM group (Fig. 3D). Western blot revealed that after supplementation with NG52, the levels of Nrf2, Ho-1 and NQO1 increased, whereas the level of Keap1 decreased (Figs. 3E and S1B). Moreover, DHE staining revealed that, in the NG52 group, the ROS levels were decreased (Figs. 3F and S1C). The IHC results revealed lower levels of 4-HNE in the placentas of the NG52 group than in those of the GDM group (Fig. 3G). These findings suggest that inhibiting PGK1 can improve HG-induced adverse pregnancy outcomes and reduce OS *in vivo*.

To investigate the role of PGK1 in OS under HG conditions, we used 20 μM CBR-470-1, a PGK1 inhibitor, in HTR8/SVneo cells. As shown in Figs. 3H and S1D, the expression of antioxidant proteins was decreased in the HG group. However, these changes were reversed when CBR-470-1 was administered. Additionally, inhibiting PGK1 restored HG-induced dysregulation of the Keap1-Nrf2 pathway. We detected the oxygen consumption rate (OCR) assay and ATP production to assess glucose-induced mitochondria impairment. We found HG impaired both basal and maximal respiration, leading to decreased ATP content (Figs. 3I, S1E, and S1F). Mitochondrial morphology was visualized using Mito Tracker staining, and mitochondrial membrane potential (MCMP) was assessed with JC-1 staining 20. Collectively, these analyses demonstrated that CBR-470-1 significantly attenuated HG-induced mitochondrial damage (Figs. 3J, 3K and S1G). The mitochondria are the major site of ROS production. Thus, we examined the intracellular and mitochondrial ROS levels using DCFH and MitoSOX staining, respectively. Both assays consistently revealed that CBR-470-1 effectively suppressed HG-induced ROS accumulation (Fig. 3L, 3M, S1H, and S1I). Given that NG52 and CBR-470-1 are both PGK1 inhibitors with distinct pharmacological profiles, we additionally evaluated the effects of NG52 *in vitro*. Our results demonstrated that NG52 treatment effectively attenuated HG-induced suppression of the Nrf2-ARE signaling pathway (Fig. S1J).

These results indicate that PGK1 inhibition alleviates GDM-induced OS and improves pregnancy outcomes by restoring Keap1-Nrf2 signaling and mitochondrial function both *in vivo* and *in vitro*.

### Effects of PGK1 on OS in HTR8/SVneo cells under HG stimulation

To further validate the role of PGK1 in HTR8/SVneo cells under hyperglycemic conditions, we established stable PGK1-knockdown HTR8/SVneo cells using lentiviral shRNA (shPGK1).

**Figure 4:**
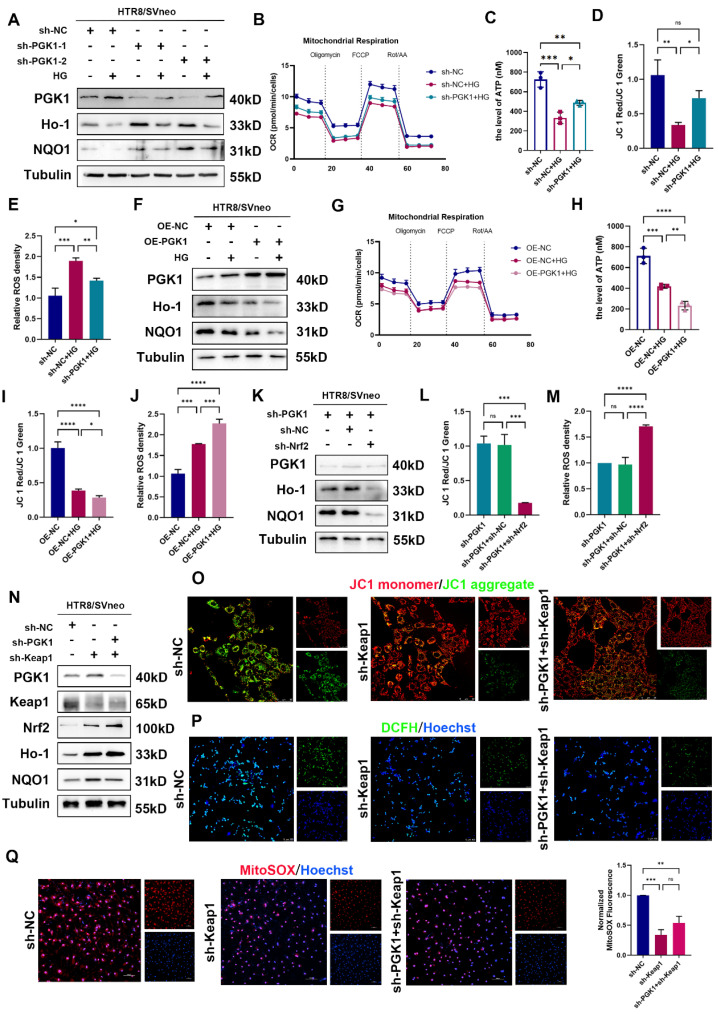
** Silencing PGK1 protects against the effects of HG on HTR8/SVneo cells by regulating Keap1-Nrf2 complex formation.** (A) Representative Western blot images of PGK1, Ho-1 and NQO1 in HTR8/SVneo cells. The cells were stably transfected with two independent PGK1-specific shRNAs (sh-PGK1-1 and sh-PGK1-2) or a nontargeting control shRNA (sh-NC), followed by treatment under normal or HG conditions. (B) OCR measurements in various groups; n = 3-5. (C) Total ATP content detection; n = 3. (D) Quantitative analysis of MCMP in the sh-NC group under normal conditions, the sh-NC group under HG stimulation, and the sh-PGK1-1 group under HG stimulation; n = 3. (E) Statistical analysis of the levels of ROS in various groups; n = 3. (F) Representative Western blot images of PGK1, Ho-1 and NQO1 in OE-PGK1 and OE-NC cells subjected to normal or HG stimulation. (G) OCR measurements in various groups; n = 3-5. (H) Total ATP content detection; n = 3. (I) Quantitative analysis of MCMP levels in various groups; n = 3. (J) Statistical analysis of the levels of ROS in various groups; n = 3. (K) Representative Western blot images and quantification of PGK1, Ho-1 and NQO1 in HTR8/SVneo cells stably transfected with sh-PGK1-1, nonsense control shRNA combined with shPGK1-1 (sh-PGK1+sh-NC), or Nrf2 shRNA combined with shPGK1-1 (sh-PGK1+sh-Nrf2). (L) Quantification of MCMP in various groups; n = 3. (M) Statistical analysis of the levels of ROS in the sh-PGK1, sh-PGK1+sh-NC, and sh-PGK1+sh-Nrf2 groups; n = 3. (N) Representative Western blot images of PGK1, Keap1, Nrf2, Ho-1, and NQO1 in HTR8/SVneo cells stably transfected with nonsense control shRNA (sh-NC), Keap1 shRNA (sh-Keap1), or sh-Keap1 combined with PGK1 shRNA (sh-Keap1+sh-PGK1). (O) Representative fluorescence images showing MCMP in the various groups; scale bar = 50 μm. (P) Representative fluorescence images showing DCFH-DA (green) staining in the sh-NC, sh-Keap1, and sh-Keap1+sh-PGK1 groups. Nuclei were counterstained with Hoechst (blue); scale bar = 100 μm. (Q) Representative fluorescence images showing MitoSOX staining (red) in HTR8/SVneo cells, and nuclei were counterstained with Hoechst (blue); scale bar = 100 μm. * represents *p* < 0.05, ** represents *p* < 0.01, *** represents *p* < 0.001, **** represents *p* < 0.0001, and ns represents *p* > 0.05.

As shown in Fig. S2A and S2B, PGK1 knockdown increased the expression of Nrf2 and phosphorylated Nrf2 (p-Nrf2) and decreased the expression of Keap1. Importantly, PGK1 knockdown effectively reversed the HG-induced suppression of the antioxidant proteins NQO1 and Ho-1 (Fig. 4A and S2C). Functional assays and immunofluorescence demonstrated that PGK1 inhibition not only ameliorated the HG-induced impairments in the OCR and ATP production (Fig. 4B, 4C and S2D) but also restored MCMP (Figs. 4D and S2E). Furthermore, both the intracellular and mitochondrial ROS accumulation induced by HG were markedly attenuated upon PGK1 knockdown (Figs. 4E, S2F, and S2G).

Next, we constructed HTR8/SVneo cell lines with stable PGK1 overexpression using lentiviruses. As shown in Fig. S3A and S3B, PGK1 overexpression decreased the levels of Nrf2 and p-Nrf2 and elevated Keap1 levels in HTR8/SVneo cells. Compared with those in vector control cells, the levels of the antioxidant proteins NQO1 and Ho-1 were further diminished in OE-PGK1 cells under HG conditions (Figs. 4F and S3C). Moreover, OCR, ATP content detection and immunofluorescence staining (JC-1, DCFH, and MitoSOX) revealed that PGK1 overexpression exacerbated HG-induced damage to mitochondrial function (Figs. 4G-4J and S3D-S3G).

Taken together, these results indicate that PGK1 inhibition protects HTR8/SVneo cells from HG-induced OS by modulating the activation of the Nrf2 signaling cascade.

### Silencing PGK1 protects against the effects of HG on HTR8/SVneo cells by regulating the Keap1-Nrf2 pathway

To demonstrate the relationship between PGK1 inhibition and Nrf2 signaling cascade activation, we constructed an Nrf2-knockdown lentivirus and a Keap1-knockdown lentivirus (Fig. S4A and S4F) and established Nrf2-deficient sh-PGK1 HTR8/SVneo cells (Fig. 4K and S4B). Strikingly, Nrf2 ablation completely abrogated the upregulation of the antioxidant proteins NQO1 and Ho-1 mediated by PGK1 knockdown. Furthermore, the functions of MCMP and ROS were measured, and the results revealed that Nrf2 knockdown abolished the protective effects induced by PGK1 knockdown in HTR8/SVneo cells under HG stimulation (Fig. 4L, 4M, S4C-E). These results suggest that Nrf2 activation is required for PGK1 inhibition-mediated protection against HG in HTR8/SVneo cells.

Our findings demonstrate that PGK1 modulates Keap1 expression (Fig. 3I) and that Keap1 methylglyoxal modification and dimerization can activate the Nrf2 cascade 11,21. To determine whether the effect of PGK1 on Nrf2 activation is caused by Keap1 degradation, we generated Keap1-knockdown and Keap1/PGK1-double-knockdown HTR8/SVneo cells stimulated with HG. Intriguingly, while Keap1 silencing alone substantially activated Nrf2 signaling, combined PGK1/Keap1 knockdown only marginally enhanced this effect (Figs. 4N and S4G). Keap1 knockdown alone significantly protected against HG-induced mitochondrial dysfunction and oxidative stress compared with sh-NC controls (Figs. 4O-4Q, S4H-4J). However, the additional knockdown of PGK1 only marginally enhanced these protective effects, suggesting that PGK1 primarily exerts its cytoprotective effect through Keap1-mediated pathways.

Additionally, the OS induced by PGK1 overexpression was partially blocked by Keap1 knockdown (Fig. S5A-S5D). These findings suggest that silencing PGK1 exerts a cytoprotective effect against HG in HTR8/SVneo cells by activating the Keap1‒Nrf2 cascade.

### Estradiol is involved in PGK1-regulated Keap1 dimer formation

**Figure 5:**
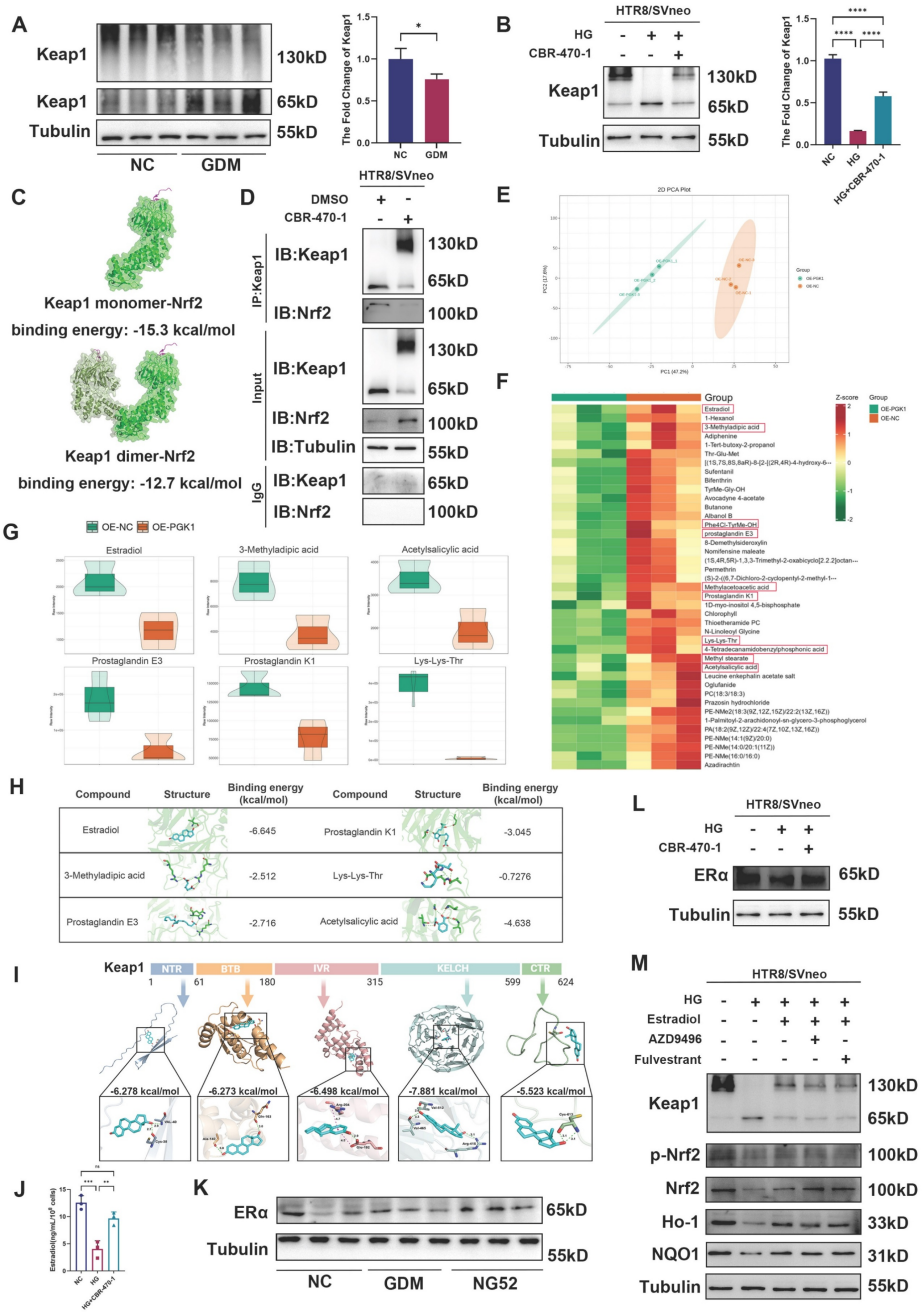
** Metabolomics-guided discovery of PGK1-regulated Keap1 dimerization modulators.** (A)Representative Western blot images and quantification of Keap1 dimers in NC and GDM human placentas; n = 3. (B) Representative Western blot images and quantification of Keap1 dimers in HTR8/SVneo cells; n = 3. (C) The predicted binding complex models of the Keap1 monomer and Keap1 dimer combined with Nrf2. (D) The interactions between Keap1 and Nrf2 with and without CBR-470-1 treatment were measured by immunoprecipitation. (E) PCA of the two groups; n = 5. (F) Heatmap of downregulated metabolites. Metabolites framed in red were further confirmed with chemical standards by matching the retention times of MSl and MS2. (G) Violin plots of selected reduced metabolites with active functional groups. (H) Schematic diagrams of the six primary metabolites screened and their binding energies with the Keap1 monomer. (I) The predicted binding complex models of the five known domains of the Keap1 protein combined with estradiol. (J) ELISA was performed to measure estradiol levels in cell culture supernatants; n = 3. (K) Representative Western blot images of ERα in the three groups of mice. (L) Representative Western blot images of ERα in HTR8/SVneo cells. (M) Representative Western blot images of Keap1, p-Nrf2, Nrf2, Ho-1 and NQO1 in HTR8/SVneo cells. * represents *p* < 0.05, ** represents *p* < 0.01, *** represents *p* < 0.001, **** represents *p* < 0.0001, and ns represents *p* > 0.05.

Previous studies have reported that PGK1 inhibition can result in the accumulation of the reactive metabolite methylglyoxal, which selectively modifies Keap1 to form a methylimidazole crosslink and cause Keap1 dimerization 11. To further investigate the mechanism by which PGK1 regulates Keap1, we used CBR-470-1 to inhibit PGK1. We first examined the expression levels of the Keap1 dimer in the placental tissues of patients and model mice. Western blot revealed that the levels of the Keap1 dimer were lower in GDM patients than in non-GDM patients (Fig. 5A) and lower in the GDM group than in the NC group, whereas inhibiting PGK1 with NG52 reversed this decrease (Fig. S6A). Compared with normal control conditions, HG stimulation decreased the level of the Keap1 dimer *in vitro*, whereas CBR-470-1 supplementation reversed this decrease in HTR8/SVneo cells (Fig. 5B).

According to the description of Keap1 dimerization in the literature 22, the full-length Keap1 structure and the dimerization structure of Keap1 were predicted by AlphaFold3. The predicted binding complex models of the Keap1 monomer and Keap1 dimer combined with Nrf2 were obtained, with binding energies of -15.3 kcal/mol and -12.7 kcal/mol, respectively (Figs. 5C and S6B). Furthermore, to verify this finding, we treated HTR8/SVneo cells with CBR-470-1 for 24 hours and harvested them for coimmunoprecipitation using a Keap1 antibody. Western blot suggested that PGK1 inhibition reduced the monomeric form of Keap1 and reduced the amount of Nrf2 pulled down (Fig. 5D). Consistent with the established mechanism in other systems 11, our study confirms that in GDM placentas, PGK1 inhibition confers protection through Keap1 dimerization-mediated Nrf2 activation and subsequent upregulation of antioxidant genes. Importantly, we further demonstrate that this pathway functionally links hyperglycemia-induced metabolic stress to placental oxidative damage in GDM.

A previous study reported that small-molecule metabolites can participate in the covalent modification of proteins and promote coupling between glucose metabolism and the Keap1-Nrf2 signaling pathway 11. To investigate the metabolic changes that affect the Keap1‒Nrf2 signaling pathway in trophoblast cells, we performed metabolomics analysis of PGK1-overexpressing cells and control cells (Figs. 5E and S6C). We screened metabolites that were significantly downregulated after PGK1 expression and identified those with high LC/MS scores (Fig. 5F). We further screened 6 metabolic substrates with reactive functionalities (Fig. 5G), each of which was subjected to molecular docking with the Keap1 monomer to calculate the binding energy. We found that estradiol had the lowest binding energy (Figs. 5H and S6D). To further elucidate the interaction between Keap1 and estradiol, we subjected each of the five known domains of Keap1 to molecular docking with estradiol. The results revealed that the Kelch domain of Keap1 had the lowest binding energy with estradiol (Fig. 5I). These findings suggest that estradiol may play a role in PGK1-mediated regulation of the Keap1-Nrf2 signaling pathway in trophoblasts. Importantly, the KEGG enrichment results suggested that various metabolism-related pathways and hormone biosynthesis pathways were affected after the overexpression of PGK1 (Fig. S6E).

To validate this mechanism, we first investigated whether PGK1 inhibition altered the expression levels of estradiol. Results showed that inhibiting PGK1 results in a significant elevation of estradiol levels (Fig. 5J). As previously reported, estradiol functions through its receptor, estrogen receptor α (ERα), which has been shown to affect Nrf2 levels through Keap1-independent mechanisms 23, 24, 25. To determine whether glucose-induced estradiol reduction mediates its effects through ERα, we quantified ERα levels in both GDM placental tissue and HG-treated HTR8/SVneo cells. Our analyses revealed no statistically significant alterations in ERα expression under these conditions (Figs. 5K, 5L, S6F and S6G). We hypothesized that estradiol exerts its effects independently of ERα under HG conditions. To test this hypothesis, we administered two distinct ERα antagonists (AZD9496 and fulvestrant) to HTR8/SVneo cells under HG stimulation. Notably, while E2 effectively attenuated HG-induced OS, this protective effect was not affected by ERα blockade, supporting our hypothesis (Figs. 5M and S6H).

These findings suggest that inhibiting PGK1 increases estradiol levels, which in turn promotes Keap1 dimerization and Nrf2 activation with a receptor-independent manner.

### HG-induced adverse pregnancy outcomes can be partly reversed by supplementation with estradiol

**Figure 6:**
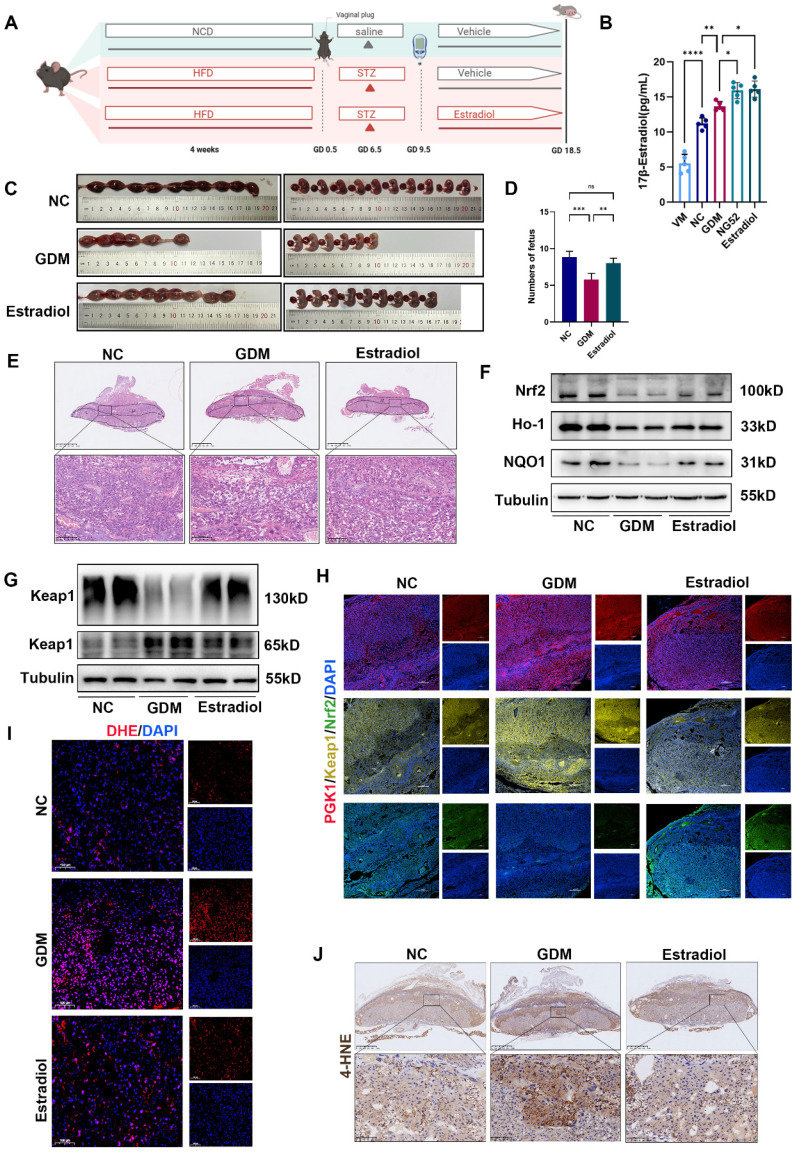
** HG-induced adverse pregnancy outcomes can be partly ameliorated by supplementation with estradiol.** (A) Schematic diagram of the grouping and treatment of the mice. (B) ELISA was performed to measure estradiol levels in mouse serum across five groups: the VM, NC, GDM, NG52, and estradiol-treated groups; n = 5. (C-D) Representative images (C) and quantification (D) of fetuses in the NC, GDM, and estradiol groups; n = 5. (E) H&E staining of mouse placentas. Up: Overview (scale bar = 1,000 μm). Down: High magnification (scale bar = 100 μm). (F) Representative Western blot images of Nrf2, Ho-1, and NQO1 in the three groups are shown. (G) Representative Western blot images of Keap1 monomers and dimers in the three groups. (H) Representative IF images of PGK1 (red), Keap1 (yellow) and Nrf2 (green) in mouse placental tissues. Nuclei were stained with DAPI (blue); scale bar = 200 μm. (I) Representative IF images of ROS (red) in mouse placental tissues. Nuclei were stained with DAPI (blue); scale bar = 100 μm. (J) Representative IHC staining of 4-HNE in NC, GDM, and estradiol-treated mouse placentas. Up: Overview (scale bar = 1 mm). Down: High magnification (scale bar = 100 μm). * represents *p* < 0.05, ** represents *p* < 0.01, *** represents *p* < 0.001, **** represents *p* < 0.0001 and ns represents *p* > 0.05.

To further elucidate the relationship between estradiol and Keap1 dimerization under hyperglycemic conditions, we administered estradiol to the GDM mouse model (Fig. 6A). On GD 18.5, compared with the GDM group, the estradiol-injected group presented a modest reduction in blood glucose levels, although this difference did not reach statistical significance (Fig. S7A).

Next, we tested the concentration of estradiol in the serum of GDM model mice by ELISA (Fig. 6B) and found that estradiol increased in the serum of GDM model mice. We administered estradiol to GDM model mice to observe whether the pregnancy outcome improved. The number of pups and H&E staining of placentas revealed that estradiol could effectively improve pregnancy outcomes and limit placental necrosis in GDM mouse placentas (Fig. 6C-6E). Western blot (Figs. 6F and S7B) revealed that estradiol supplementation increased the levels of Nrf2, Ho-1 and NQO1 and decreased the level of Keap1. Immunofluorescence analysis demonstrated that estradiol treatment primarily regulates the Keap1-Nrf2 signaling pathway in the placental middle junctional zone. (Fig. 6H). Moreover, supplementation with estradiol promoted Keap1 dimerization in the placentas of GDM mice (Figs. 6G and S7C). IF of DHE and IHC of 4-HNE staining (Figs. 6I, 6J, and S7D) revealed that estradiol ameliorated OS in the placentas of GDM mice.

Notably, we observed increased apoptotic signaling (Bax/Bcl-2 ratio) in GDM placentas, which was significantly reduced by estradiol treatment (Fig. S7E). Consistently, *in vitro* studies showed that HG elevated apoptosis in trophoblasts, and estradiol supplementation effectively suppressed this effect (Fig. S7F). These findings validate our hypothesis that exogenous estradiol administration alleviates adverse HG effects.

Taken together, these findings suggest that supplementation with estradiol can improve HG-induced adverse pregnancy outcomes and reduce OS *in vivo* and *in vitro*.

## Discussion

It is well known that GDM is associated with increased OS levels, which are due to excessive ROS generation and impaired elimination of free radicals, resulting in a mismatch between ROS production and the cellular antioxidant capacity 26, 27. Consistent with previous findings, in this study, we examined ROS levels in GDM patients and found that, compared with those in normal placentas, the levels of ROS production in GDM placentas were greater. Hyperglycemic environment leads to excessive production of ROS, which may be attributed to HG-induced blockage of the mitochondrial electron transfer chain, causing massive accumulation of ROS 28. Our findings further confirmed the finding that HG stimulation can reduce mitochondrial basal respiration and maximal respiration, leading to a decrease in ATP content. Furthermore, we observed damaged mitochondrial membrane potential under HG stimulation. In addition, the expression of the antioxidant proteins Ho-1 and NQO1 was suppressed in GDM patients, a mouse model, and in HTR8/SVneo cells stimulated with HG. An imbalance in redox homeostasis results in embryonic and fetal exposure to the harmful effects of OS, which increases miscarriages and fetal malformations 27. We found that GDM mice presented more severe negative pregnancy outcomes, with increased resorption rates and reduced fetal numbers. In the GDM group, the middle junctional zone of the placenta had larger necrotic and infarctional areas. This finding may provide a mechanism for the poor pregnancy outcomes of GDM at the placental level since the placenta is required to meet the respiratory and nutritional requirements of the fetus 29.

Given that GDM is often accompanied by glucose metabolic disorders, it is essential for clinicians to explore novel targets to restore abnormal metabolic status. Here, our work revealed cross-talk between the glycolysis pathway and the OS pathway in GDM patients. Numerous studies have indicated that PGK1 overexpression in tumorigenesis is associated with the upregulation of glycolysis and is related to OS. Inhibition of PGK1 can block tumor proliferation, suppress glycolysis, and exert anti-inflammatory and antioxidant effects 21, 30. Here, we found that PGK1 protein levels were greater in the placentas of GDM pregnancies than in those of normal pregnancies. Consistent results were observed in the placentas of GDM mice and HG-stimulated HTR8/SVneo cells. *In vitro*, inhibiting PGK1 with the PGK1 inhibitor CBR-470-1 reduced ROS production and increased ATP content. In addition, PGK1 overexpression had the opposite effect; compared with HG stimulation alone, PGK1 overexpression decreased antioxidant protein levels and impaired mitochondrial function. Furthermore, our *in vivo* results demonstrated that NG52 treatment improved pregnancy outcomes and alleviated OS in GDM mice. These results suggest that targeting metabolism may be a novel therapeutic strategy for GDM treatment.

Numerous studies have demonstrated a protective role of Nrf2-ARE signaling pathway activation in GDM 18, 31. Nrf2, Ho-1, and NQO1 expression in human placental tissue was markedly lower in GDM patients than in normal patients, and the same result was obtained in the GDM mouse model. Keap1, which is a master negative regulator of Nrf2, has reactive cysteine residues that function as electrophile sensors of reactive species 28. Upon exposure to oxidative and electrophilic stress, Keap1 cysteine oxidation decreases Nrf2 ubiquitination and increases its nuclear translocation and activation 33, 34. However, accumulating evidence has demonstrated that Nrf2 is inactivated in certain pathological conditions, such as diabetes 35. In this study, we observed an imbalance in pro-oxidant/antioxidant homeostasis in the placentas of women with GDM, especially in those with PGK1 overexpression, and these disorders were more significant. Compared with those in the GDM model, there was a larger necrotic and infarcted area in the middle junction of the placenta. One plausible explanation is that cell damage caused by sustained hyperglycemia is amplified and sustained due to its failure to correct excessive levels of glucose and ROS 36. This imbalance in redox homeostasis causes increased Keap1 expression and accelerated NRF2 degradation in an HG environment 36, 37.

Regarding the relationship between PGK1 and the Keap1-Nrf2 signaling pathway, recent studies have reported that PGK1 is an upstream regulator of Keap1-Nrf2 9,11. Therefore, we examined the link between PGK1 and the Keap1‒Nrf2 complex and found that PGK1 inhibition or silencing resulted in a significant increase in Nrf2 abundance and a decrease in Keap1 monomers. Silencing Keap1 mimicked the effects of PGK1 inhibition, whereas supplementing silenced Nrf2 eliminated the protective effects of PGK1 inhibition. These results indicate that PGK1 modulates OS by regulating the Keap1‒Nrf2 pathway. In addition, we further examined the levels of the Keap1 dimer and monomer in GDM placentas and found that the Keap1 dimer was reduced in GDM placental tissue and that HG stimulation decreased the expression of Keap1 dimers in HTR8/SVneo cells. Moreover, docking analysis provided direct evidence that Keap1 dimerization decreased its ability to bind to Nrf2. Therefore, we hypothesize that PGK1 inhibition protects against GDM by increasing Keap1 dimer formation. Our data support this hypothesis; in our system, NG52-mediated PGK1 inhibition significantly reduced placental OS, accompanied by an increase in Keap1 dimerization and improved STZ-induced pregnancy outcomes in GDM mice.

Keap1 dimerization can be regulated by metabolic substrates with reactive functionalities 14. In our PGK1 overexpression model, we found that the level of estradiol, the most bioactive endogenous estrogen 38, significantly decreased. The plasma concentration of estradiol is consistently elevated during pregnancy, peaking in late pregnancy 39. Recent studies have shown that supplementation with estradiol can inhibit inflammation and restore mitochondrial function to mitigate OS 40, 41. Although Liebmann et al. reported that estradiol has protective effects on glucose-stimulated insulin secretion and improves GDM progression 42, the effect of estradiol on the GDM placenta has not been clearly defined. Our study demonstrated that estradiol administration significantly enhances pregnancy outcomes, as evidenced by reduced embryo resorption rates and increased fetal numbers. This is most likely attributable to a decrease in the necrotic and infarctional areas of the middle junctional zone in the placenta of GDM mice in the estradiol treatment group. Mechanistically, the protective effect of estradiol on the placenta may be attributed to the activation of the Nrf2-ARE pathway; following supplementation with estradiol, antioxidant proteins increase and ROS production decreases. Furthermore, our molecular docking study confirmed that, among the top 6 most strongly downregulated metabolites, estradiol had the highest affinity for the Keap1 monomer. Notably, supplementation with estradiol was accompanied by an increase in Keap1 dimerization and Nrf2 accumulation. Therefore, our results indicate that estradiol mediates the PGK1-mediated regulation of Keap1 dimerization in the GDM placenta. In addition, a previous study reported that the serum estradiol level was increased in a New Zealand obese (NZO)-induced GDM mouse model, whereas another GDM cohort study reported that, in GDM patients, the levels of estradiol were significantly lower than those in NC patients 38, 42. Whether the serum estradiol level increases or decreases in GDM patients remains unclear. In our STZ-induced GDM models, we detected elevated serum estradiol levels in the GDM group, and supplementation with a PGK1 inhibitor further increased estradiol levels. This may be because, in GDM, increased estradiol levels partially counteract hyperglycemia-induced metabolic disorders but remain insufficient. Notably, we observed increased apoptotic signaling (Bax/Bcl-2 ratio) in GDM placentas, which was significantly reduced by exogenous estradiol treatment. Consistently, *in vitro* studies showed that high glucose elevated apoptosis in trophoblasts, and estradiol supplementation effectively suppressed this effect. Furthermore, when we administered a PGK1 inhibitor to GDM mice, the production of sufficient amounts of estradiol protected the placenta from HG-induced OS. However, the mechanism by which PGK1 regulates the levels of estradiol, a glycolytic metabolic enzyme that regulates sex hormones, remains to be further studied. A previous study reported that follicle-stimulating hormone (FSH) can regulate PGK1 expression and increase glycolysis 43. Glycolysis, as the primary source of energy metabolism substrates for oocytes in follicular granulosa cells, plays an important role in the development of ovarian follicles 44. Therefore, we hypothesize that during the pathological process of GDM, increased glycolysis causes a decrease in FSH through negative feedback. Because the production of estradiol can be regulated by FSH 45, the reduced FSH further causes a decrease in estradiol levels, particularly when PGK1 is overexpressed.

Estradiol plays a crucial role in maintaining glucose metabolic homeostasis, particularly in counteracting hyperglycemic conditions. Studies have demonstrated that during the pathological progression of diabetes, estradiol levels are decreased, and estradiol supplementation can improve β-cell function by promoting β-cell proliferation 46, 47 and exerting antiapoptotic effects 48. Similarly, Nrf2 deficiency during pregnancy leads to increased β-cell oxidative stress, increased apoptosis, and impaired proliferative capacity 49. Furthermore, in GDM, previous studies have demonstrated that estradiol levels are lower than those in normal pregnancies 50, 51, and estradiol supplementation can alleviate β-cell oxidative stress by upregulating NRF2 expression 49. This result is consistent with our study showing that in the placenta, estradiol supplementation can reduce OS in GDM through the activation of the Nrf2-ARE signaling pathway. Notably, during this process, the ERα expression level did not significantly change in the GDM placenta, and PGK1 inhibition had no apparent effect on ERα expression. Importantly, even under conditions of ERα inhibition, estradiol supplementation could still upregulate the Nrf2-ARE signaling pathway. This finding aligns with Schaufelberger's report that estradiol exerts its effects through receptor-independent mechanisms, which are mediated by its endogenous metabolite 2-methoxyestradiol 52. However, studies have indicated that estradiol can also act through its receptors to regulate the Keap1‒Nrf2 pathway, where estradiol competitively binds to Keap1 to reduce Nrf2 degradation 53. Although previous research has shown that estradiol can regulate NRF2 through Keap1-independent mechanisms, including PI3K pathway activation 54, 55 or direct binding to the NRF2 promoter region 56, our study revealed that, among PGK1-related reactive metabolites, estradiol exhibited the highest binding affinity for Keap1 and promoted Keap1 dimerization, thereby increasing Nrf2 levels.

The present study has several limitations. First, we demonstrated that inhibiting PGK1 protected GDM mice by increasing estradiol levels, promoting Keap1 dimerization, activating the Nrf2-ARE pathway, and ultimately restoring mitochondrial function to mitigate OS in the placenta (Graphical Abstract). However, although we reported that PGK1 could regulate estradiol levels, the exact mechanism still needs further investigation, especially considering that PGK1 not only functions as a metabolic enzyme to regulate glycolysis metabolism but also functions as a protein kinase to regulate mitochondrial function and autophagy initiation 57. Second, GDM is triggered mainly by impaired insulin action and β-cell dysfunction 58. Although we demonstrated that both supplementation with a PGK inhibitor and supplementation with estradiol had protective effects on placental and pregnancy outcomes in women with GDM, whether these protective effects are dependent on the recovery of β-cell function or independent needs further investigation.

## Conclusion

In conclusion, the present study demonstrated that inhibiting PGK1 has protective effects on GDM placenta and pregnancy outcomes by increasing estradiol levels, promoting Keap1 dimerization, activating the Nrf2-ARE pathway, restoring mitochondrial function, and ultimately mitigating OS in GDM placenta. Importantly, our findings reveal a novel therapeutic paradigm for GDM management, wherein glycolytic metabolism modulation serves as a pivotal regulator of intracellular redox homeostasis.

## Supplementary Material

Supplementary methods and figures.

## Figures and Tables

**Table 1:** Clinical characteristics

Characteristics	NC (n=16)	GDM (n =16)	P
**Maternal**			
Age, years	28.50±2.76	30.56±3.50	0.074
OGTT, mmol/L			
Fasting	4.44±0.35	4.85±0.66	0.036
1 h	7.46±1.24	10.21±1.10	<0.001
2 h	6.54±0.49	8.25±1.06	<0.001
BMI, kg/m^2^	23.23±0.84	25.94±1.15	0.279
Parity	1.63±0.62	1.31±0.48	0.121
Gestational age, days	276.94±7.17	278.00±3.43	0.579
**Neonatal**			
Sex, n%			1.000
Boy	8 (50.00)	8 (50.00)	
Girl	8 (50.00)	8 (50.00)	
Delivery status, n%			1.000
Vaginal	12 (75.00)	13 (81.25)	
Cesarean	4 (25.00)	3 (18.75)	
Birth-weight, g	3,175.00±338.76	3,202.5±303.44	0.811

The data are shown as the means ± SDs.

**Table 2:** Sequences used in the study

Genes	Target DNA sequence
PGK1 shRNA-S1	GGACAAGCTGGACGTTAAAGG
PGK1 shRNA-S2	GCTGTCCCAAGCATCAAATTC
Keap1 shRNA	GCGAATGATCACAGCAATGAA
Nrf2 shRNA	CCGGCATTTCACTAAACACAA
