# Supplementary material for: PGK1 Regulates Oxidative Stress in Gestational Diabetes Mellitus through the Estradiol-Keap1-Nrf2 Pathway
Source: Int J Biol Sci. 2025 Aug 25;21(12):5496–513. doi: 10.7150/ijbs.113728 (PMC12435576; doi:10.7150/ijbs.113728)
Supplement: Supplementary file 1 — Supplementary methods and figures. [file ijbsv21p5496s1.pdf]

Supplemental Information

Table of Contents

1) Expanded Methods and Materials

2) Reference for supplemental information

3) Supplemental Figures

1. Expanded Methods and Materials

1.1 Immunohistochemistry, immunofluorescence, and H&E staining

As previously described [1], the first step involved dewaxing and hydrating placental sections from humans or mice.

For immunohistochemistry, we performed heat-induced antigen retrieval with sodium citrate buffer. Endogenous peroxidase was inactivated with 3% hydrogen peroxide for 20 min. Nonspecific binding sites were blocked with 1% goat serum albumin at room temperature for 20 min. After incubation with a PGK1 antibody (Proteintech, USA) or a 4-hydroxynonenal (4-HNE) antibody (Abcam, USA) overnight at 4 °C, the sections were incubated with goat anti-rabbit IgG-HRP (ImmunoWay, USA) at room temperature for 30 min. After washing with TBS, the sections were stained and counterstained with DAB working reagent and hematoxylin, respectively.

Immunofluorescence was performed as previously reported [2], and the PGK1 level in the placentas of humans and the PGK1, Bcl-2 (Abcam, USA) and Bax (Abcam, USA) levels in the placentas of mice were measured. The stained tissues were viewed using a confocal fluorescence microscope (Leica, Germany). The quantification of the immunofluorescence staining images was performed using ImageJ software.

For H&E staining, the nucleus was stained blue by hematoxylin (Beyotime, China), and the cytoplasm was stained red by eosin. After dehydration, a microscope was used to examine the sections.

1.2 RNA-sequencing

RNA was isolated from the placental tissues of three patients diagnosed with GDM (without other pregnancy complications and underlying diseases) and three normal controls (without any pregnancy complications and underlying diseases). These RNA specimens were forwarded to Berry Genomics Co., Ltd., located in Beijing, China, for the creation of cDNA libraries and subsequent sequencing, which was carried out on the Illumina NovaSeq 6000 system. The initial sequencing outputs were matched against the human reference genome using Hisat2 version 2.0.5. The enumeration of reads corresponding to each gene was

accomplished through computational means. Following this, gene expression levels were quantified using the FPKM method. The DESeq2 R package, specifically version 1.20.0, was employed to analyse differential expression, with genes exhibiting a p-value below 0.05 being identified as differentially expressed (DEGs).

1.3 Seahorse assay

After stimulation for 24 hours, HTR8/SVneo cells were seeded in an XFe96 cell culture microplate with 1.5×10^5 cells per well, and 3 to 5 replicates were performed for each group. The extracellular acidification rate (ECAR) and oxygen consumption rate (OCR) of HTR8/SVneo cells were measured using an oxygen-controlled XFe96 extracellular flux analyzer (Agilent, USA) according to the manufacturer's instructions as previously reported [3]. For the ECAR, rotenone/antimycin A (0.5 μM) and 2-deoxyglucose (50 mM) were added to the cells in sequence. For the OCR, oligomycin (1.5 μM), FCCP (1 μM) and rotenone/antimycin A (0.5 μM) were added to the cells in sequence. Basal glycolysis or basal respiration was measured under basal conditions before any injection. The compensatory glycolysis rate was the maximum glycoPER after Rot/AA injection. The maximal respiratory capacity was calculated as the OCR after the addition of FCCP minus the OCR after the addition of oligomycin. Cell images and numbers were acquired for normalization using a Cytation5 imaging reader (BioTek, USA).

1.4 Mitochondrial Function and Oxidative Stress Assessment

Mitochondrial morphology was visualized using MitoTracker staining (Invitrogen, USA). The mitochondrial membrane potential (MCMP), reactive oxygen species (ROS) and mitochondrial superoxide of HTR8/SVneo cells were detected as previously reported by DCFH-DA fluorescent probe (Beyotime, China), JC-1 Kit (Beyotime, China) and MitoSOX mitochondrial superoxide indicator (Solarbio, China), respectively, according to the manufacturer's instructions. The stained cells were viewed using a confocal fluorescence microscope (Leica, Germany). For tissue ROS measurement, human or mouse placentas were frozen and sectioned into 6 μm sections. After being incubated with 10 μM DHE staining solution at room temperature in the dark for 30 min, the sections were sealed with an anti-fluorescence quenching agent (Beyotime, China). Images were captured using a fluorescence microscope (Zeiss, Germany). The quantification of the images was analyzed using ImageJ software.

1.5 ATP content quantification

ATP content was quantified using a Beyotime (China) kit. After removing the medium, 200 μL of lysis buffer was added to each well of the 6-well plate and pipetted repeatedly to ensure

complete cell lysis. The buffer was then centrifuged at 12000 × g at 4 °C to collect the supernatants. A volume of 100 μl of diluted ATP test solution was added to the 96-well plate and left at room temperature for 5 minutes. Then, 20 μL of sample or standard was added and quickly mixed. The RLU values were measured with a luminometer (TECAN, Switzerland). The protein concentration was detected by a BCA assay kit (Beyotime, China) for normalization.

1.6 Western blot

The placental tissues were lysed in 500 μL of RIPA lysis buffer supplemented with protease and phosphatase inhibitors (Beyotime, China) and ground using a freezer mill (Luka, China). The protein concentration was quantified using a BCA assay kit (Beyotime, China) to ensure that 10 μg of protein was separated by SDS-polyacrylamide gel electrophoresis. Proteins were then transferred onto polyvinylidene difluoride membranes (Millipore, USA). The primary detection antibodies used in our study were anti-β-tubulin (1:10000; Affinity, USA), anti-Nrf2 (1:1000; Proteintech, USA), anti-Keap1 (1:1000; Proteintech, USA), anti-Keap1 (1:200; Santa Claus, USA), anti-PGK1 (1:1000; Proteintech, USA), anti-NQO1 (1:1000; Proteintech, USA), anti-Ho-1 (1:1000; Proteintech, USA), anti-p-Nrf2 (1:1000; Bioss, China) and anti-ERα (1:1000; Proteintech, USA). Dimerized Keap1 was detected by nondenaturing SDS–PAGE [4]. The secondary antibodies used were goat anti-rabbit and anti-mouse IgG-HRP (1:5000, Fudebio, China). Western blot images were quantified using ImageJ software.

1.7 RNA isolation and RT-qPCR

Total RNA was extracted using TRIzol Reagent (TIANGEN, Germany) and quantified using a Nanodrop instrument (ThermoFisher Scientific, USA). 1 μg of total RNA was reverse transcribed to cDNA with a HiScript Q RT SuperMix for qPCR kit (Vazyme, China), and real-time quantitative PCR was performed with ChamQ SYBR qPCR Master Mix (Vazyme, China). β-Actin was used to normalize mRNA expression, and the $2^{-\Delta\Delta CT}$ method was used to analyze the relative mRNA expression. The primers used in this study were as follows:

**Genes**	**Target DNA sequence**
human PGK1-forward	5′- CCGCTTTCATGTGGAGGAAGAAG -3′
human PGK1-reverse	5′- CTCTGTGAGCAGTGCCAAAAGC -3′
human HK1-forward	5′- CTGCTGGTGAAAATCCGTAGTGG -3′
human HK1-reverse	5′- GTCCAAGAAGTCAGAGATGCAGG -3′
human PFKL-forward	5′- AAGAAGTAGGCTGGCACGACGT -3′
human PFKL-reverse	5′- GCGGATGTTCTCCACAATGGAC -3′

human GAPDH-forward	5′- GTCTCCTCTGACTTCAACAGCG -3′
human GAPDH-reverse	5′- ACCACCCTGTTGCTGTAGCCAA -3′
human PKM2-forward	5′- ATGGCTGACACATTCCTGGAGC -3′
human PKM2-reverse	5′- CCTTCAACGTCTCCACTGATCG -3′
human LDHA-forward	5′- GGATCTCCAACATGGCAGCCTT -3′
human LDHA-reverse	5′- AGACGGCTTTCTCCCTCTTGCT -3′
human β-Actin-forward	5′- CACCATTGGCAATGAGCGGTTC -3′
human β-Actin-reverse	5′- AGGTCTTTGCGGATGTCCACGT -3′

1.8 Coimmunoprecipitation (Co-IP)

The Co-IP assay was performed using Co-IP kits (Absin, China) according to the manufacturer's recommended protocol. After being harvested and washed, HTR8/SVneo cells were subsequently lysed with 500 μL of precooled lysis buffer and then scraped off for ultrasound fragmentation. The lysate was centrifuged at 14,000 × g for 10 min at 4 °C, with 100 μL of supernatant left as an input control. A total of 500 μg of extract was incubated with 10 μg of Keap1 antibody (and IgG as a negative control) at 4 °C overnight, and then 5 μL of Protein A and 5 μL of Protein G were added to the mixture and mixed for 2 hours at 4 °C. After extensive washing with 1× Wash Buffer, the immunoprecipitates was used in subsequent assays.

1.9 Metabolomics study and data analysis

A HTR8/SVneo cell line that stably overexpresses PGK1 and a cell line infected with blank vector lentivirus were established for metabolomic analysis by MetWare Co., Ltd. (China). The cells were planted into 10 cm dishes, collected at cell densities of 90% confluence, and then stored at -80 °C. The cells were freeze-thawed three times and then centrifuged to obtain the supernatant used for the two LC/MS methods. One aliquot was analyzed under positive ion conditions, and another aliquot was analyzed under negative ion conditions. The data were acquired in information-dependent acquisition (IDA) mode using Analyst TF 1.7.1 Software (Canada) and then converted into mzXML format by ProteoWizard software. Peak extraction, peak alignment, and retention time correction were performed by the XCMS program. The "SVR" method was used to correct the peak area. The peaks with detection rates lower than 50% in each group of samples were discarded. Afterward, metabolic identification information was obtained by searching the laboratory's self-built database, integrated public database, AI database and metDNA.

1.10 Enzyme-linked immunosorbent assay (ELISA) assays

Serum levels of estradiol in mice and cell culture supernatants were measured using specified ELISA kits (MEIMIAN, China). Standards and samples were added to ELISA plates and incubated at 37°C for 30 min. After five washes, horseradish peroxidase-conjugate reagent was added and incubated at 37°C for 30 min. Following five more washes, chromogen solution was incubated at 37°C for 10 min, then stopped. Optical density (OD) was read at 450 nm. Sample concentrations were determined from a standard curve plotted using the standards' ODs.

1.11 Flow cytometry

Cell apoptosis was examined using a FITC Annexin V Apoptosis Detection Kit I (Biosciences, USA). Briefly, HTR8/SVneo cells were washed twice with cold PBS and then resuspended in 1× binding buffer at a concentration of 1 × 10^6 cells/mL, after which 100 μL of the solution was transferred to a 5 mL culture tube, and 5 μL of FITC Annexin V and 5 μL of PI were added. After incubation in the dark at room temperature for 15 min, 400 μL of 1× binding buffer was added to each tube. The cells were analyzed by flow cytometry within 1 hour by a Cyan ADP 9C flow cytometer (Beckman, France).

1.12 Computational binding simulation

The AlphaFold3 online server was used to predict the tertiary structure of the protein, and the PubChem online website was used to search for structural information on small molecules and to download SDF files, which were then converted to OpenBable files to obtain PDB files. AutoDock 4.2 and AutoDock Vina were used for molecular docking simulation analysis and for obtaining binding energy data. PyMOL is used to visualize hydrogen bond interactions [5].

2. Reference

1. Hu H, Jiang J, Chen Q, et al. Cyclophilin A inhibits trophoblast migration and invasion in vitro and vivo through p38/ERK/JNK pathways and causes features of preeclampsia in mice. Life Sci. 2020 Nov 15;261:118351.

2. Zhao H, Xian G, Zeng J, et al. Hesperetin, a Promising Dietary Supplement for Preventing the Development of Calcific Aortic Valve Disease. Antioxidants (Basel). 2022 Oct 24;11(11):2093.

3. Wu H, Zhao X, Hochrein SM, et al. Mitochondrial dysfunction promotes the transition of precursor to terminally exhausted T cells through HIF-1α-mediated glycolytic reprogramming. Nat Commun. 2023 Oct 27;14(1):6858.

4. Bollong MJ, Lee G, Coukos JS, et al. A metabolite-derived protein modification integrates glycolysis with KEAP1-NRF2 signalling. Nature. 2018;562(7728):600-604.

5. Eberhardt J, Santos-Martins D, Tillack AF, et al. AutoDock Vina 1.2.0: New Docking Methods, Expanded Force Field, and Python Bindings. J Chem Inf Model. 2021 Aug 23;61(8):3891-3898.

3. Supplemental Figures

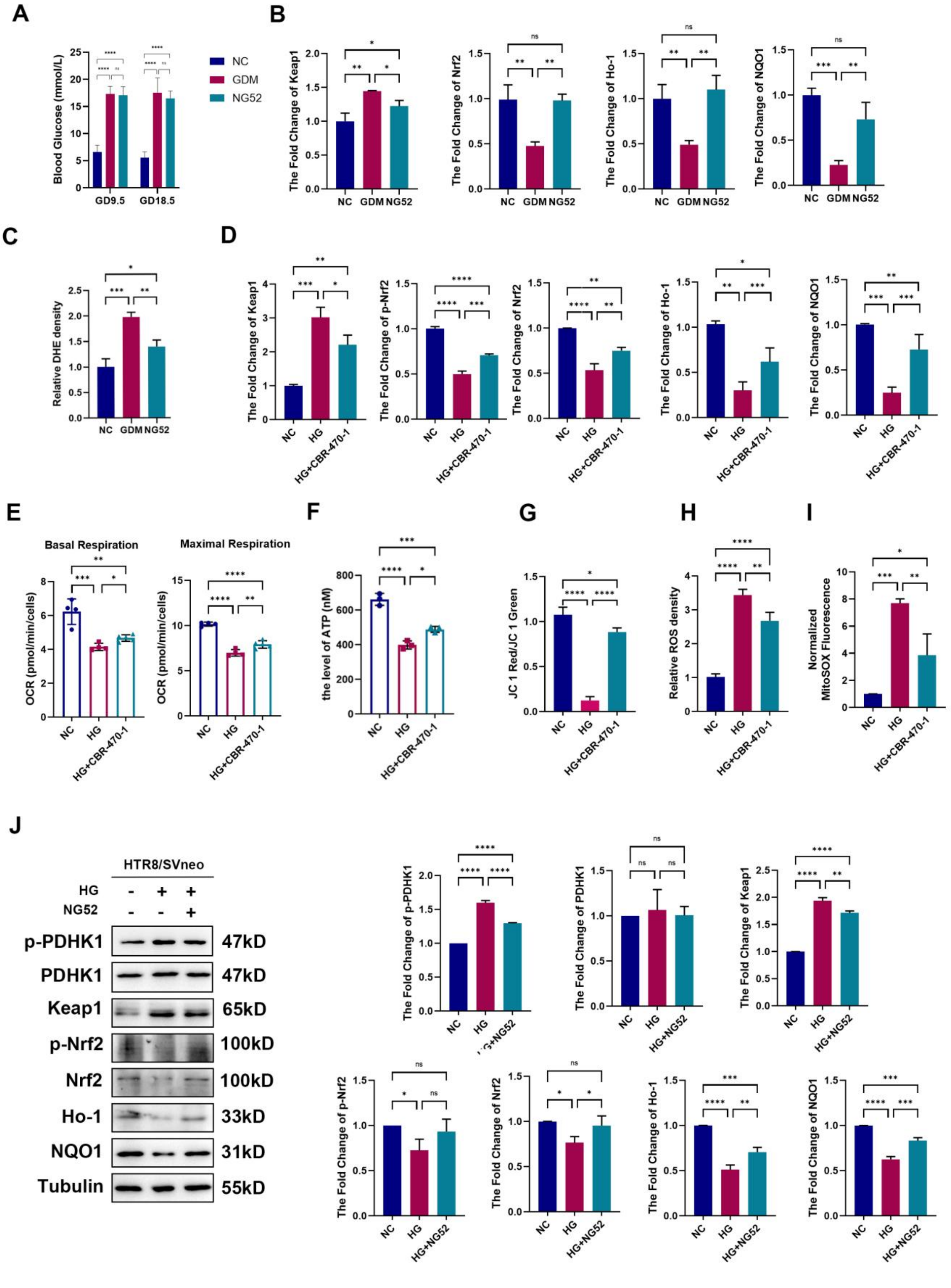

Supplementary Fig. 1 | Inhibiting PGK1 protects against the effects of high glucose (HG) on HTR8/SVneo cells.

(A) Blood glucose of mice; n = 5.

(B) Quantification of Keap1, Nrf2, Ho-1 and NQO1 levels in mouse placentas; n = 3.

(C) Quantitative analysis of ROS in mouse placental tissues; n = 3.

(D) Quantification of the levels of Keap1, p-Nrf2, Nrf2, Ho-1 and NQO1 in HTR8/SVneo cells; n = 3.

(E) Basal respiration was measured under basal conditions, and the maximal respiratory capacity was calculated as the OCR after adding FCCP minus the OCR after adding oligomycin; n = 4-5.

(F) Total ATP content detection; n = 3.

(G) Quantification of MCMP; n = 3.

(H) Quantitation of DCFH-DA intensity in HTR8/SVneo cells; n = 3.

(I) Quantification of MitoSOX Red fluorescence; n = 3.

(J) Representative images and quantification of the protein levels of p-PDHK1, PDHK1, Keap1, p-Nrf2, Nrf2, Ho-1 and NQO1 in HTR8/SVneo cells; n = 3.

* represents $p < 0.05$, ** represents $p < 0.01$, *** represents $p < 0.001$, **** represents $p < 0.0001$ and ns represents $p > 0.05$.

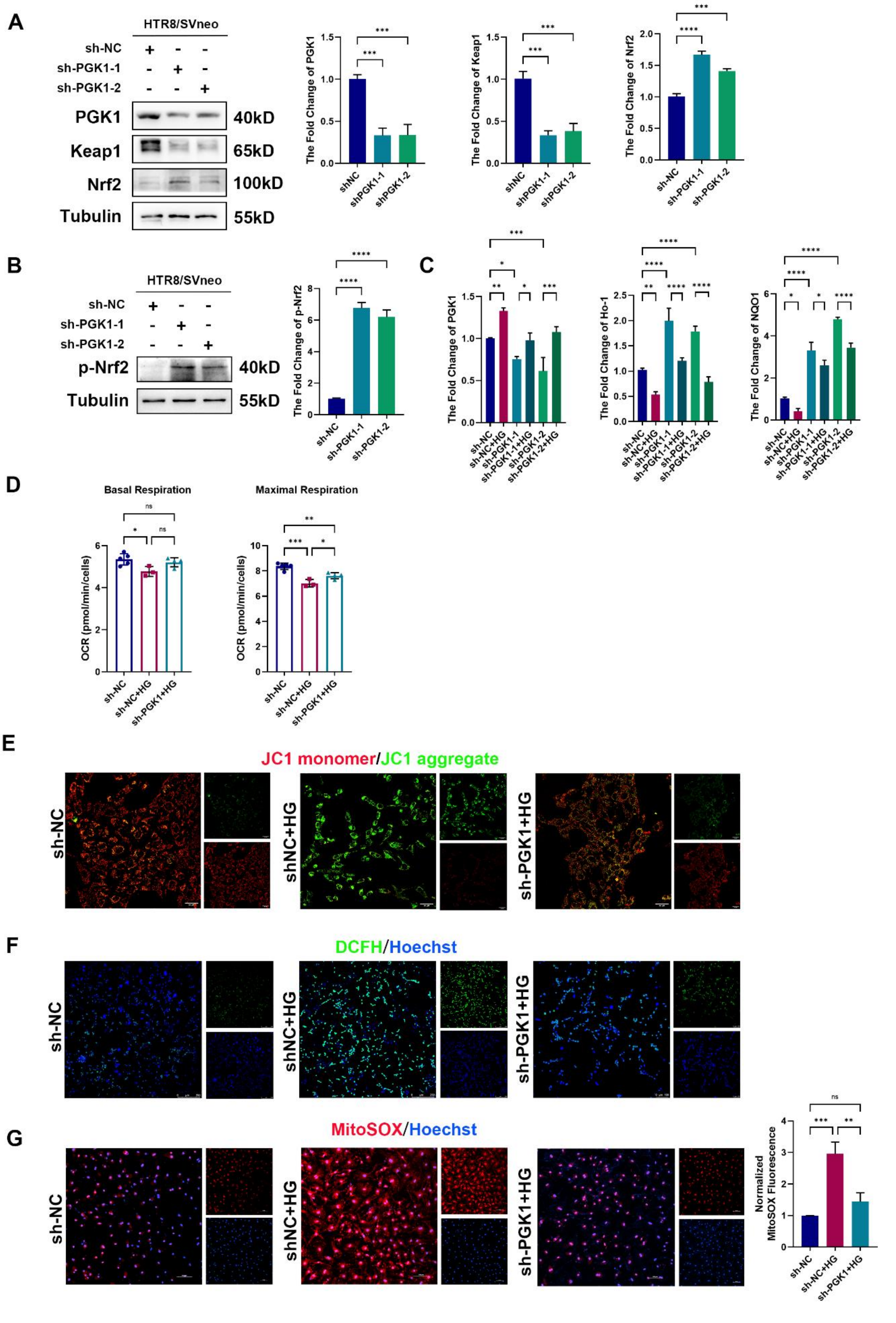

Supplementary Fig. 2 | PGK1 knockdown reduces oxidative stress (OS) caused by HG stimulation in HTR8/SVneo cells.

(A) Representative Western blot images and quantitative data showing the protein levels of PGK1, Keap1, and Nrf2 in HTR8/SVneo transfected with sh-NC, sh-PGK1-1, and sh-PGK1-2; n = 3.
(B) Representative Western blot images and quantitative data showing the protein levels of p-Nrf2 in the various groups; n = 3.
(C) Quantitative data of the protein levels of PGK1, Ho-1 and NQO1 in the sh-NC, sh-PGK1-1, and sh-PGK1-2 treated under normal or HG conditions; n = 3.
(D) Basal respiration and the maximal respiratory capacity of the OCR assay; n = 3 - 5.
(E) Representative fluorescence images showing MCMP in the various groups; scale bar = 50 μm.
(F) Representative fluorescence images showing ROS levels in the various groups; scale bar = 250 μm.
(G) Representative fluorescence images and quantification of MitoSOX staining (red) in HTR8/SVneo cells, and Nuclei were counterstained with Hoechst (blue); scale bar = 100 μm; n = 3.
* represents $p < 0.05$, ** represents $p < 0.01$, *** represents $p < 0.001$, **** represents $p < 0.0001$ and ns represents $p > 0.05$.

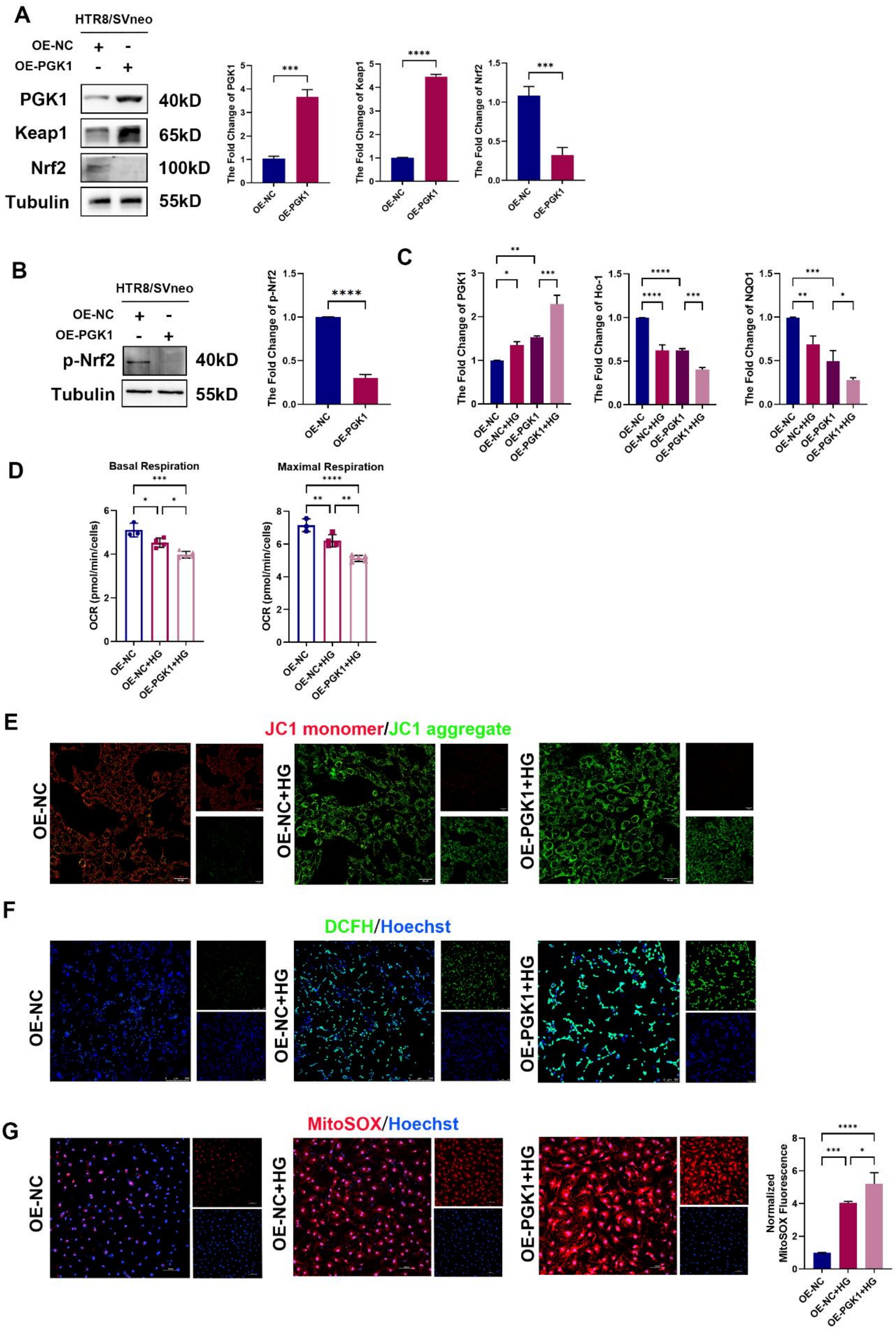

Supplementary Fig. 3 | Increased PGK1 aggravates OS caused by HG stimulation in HTR8/SVneo cells.

(A) Representative Western blot images and quantitative data showing the protein levels of PGK1, Keap1, and Nrf2 in the OE-NC and OE-PGK1 groups; n = 3.

(B) Representative Western blot images and quantitative data showing the protein levels of p-Nrf2 in the OE-NC and OE-PGK1 groups; n = 3.

(C) Quantitative data of the protein levels of PGK1, Ho-1 and NQO1 in the OE-NC and OE-PGK1 groups treated under normal or HG conditions; n = 3.

(D) Basal respiration and the maximal respiratory capacity of the OCR assay; n = 3 - 5.

(E) Representative fluorescence images showing MCMP levels in the various groups; scale bar = 50 μm.

(F) Representative fluorescence images showing ROS levels in the various groups; scale bar = 250 μm.

(G) Representative fluorescence images and quantification of MitoSOX staining (red) in HTR8/SVneo cells, and Nuclei were counterstained with Hoechst (blue); scale bar = 100 μm; n = 3.

* represents $p < 0.05$, ** represents $p < 0.01$, *** represents $p < 0.001$, **** represents $p < 0.0001$.

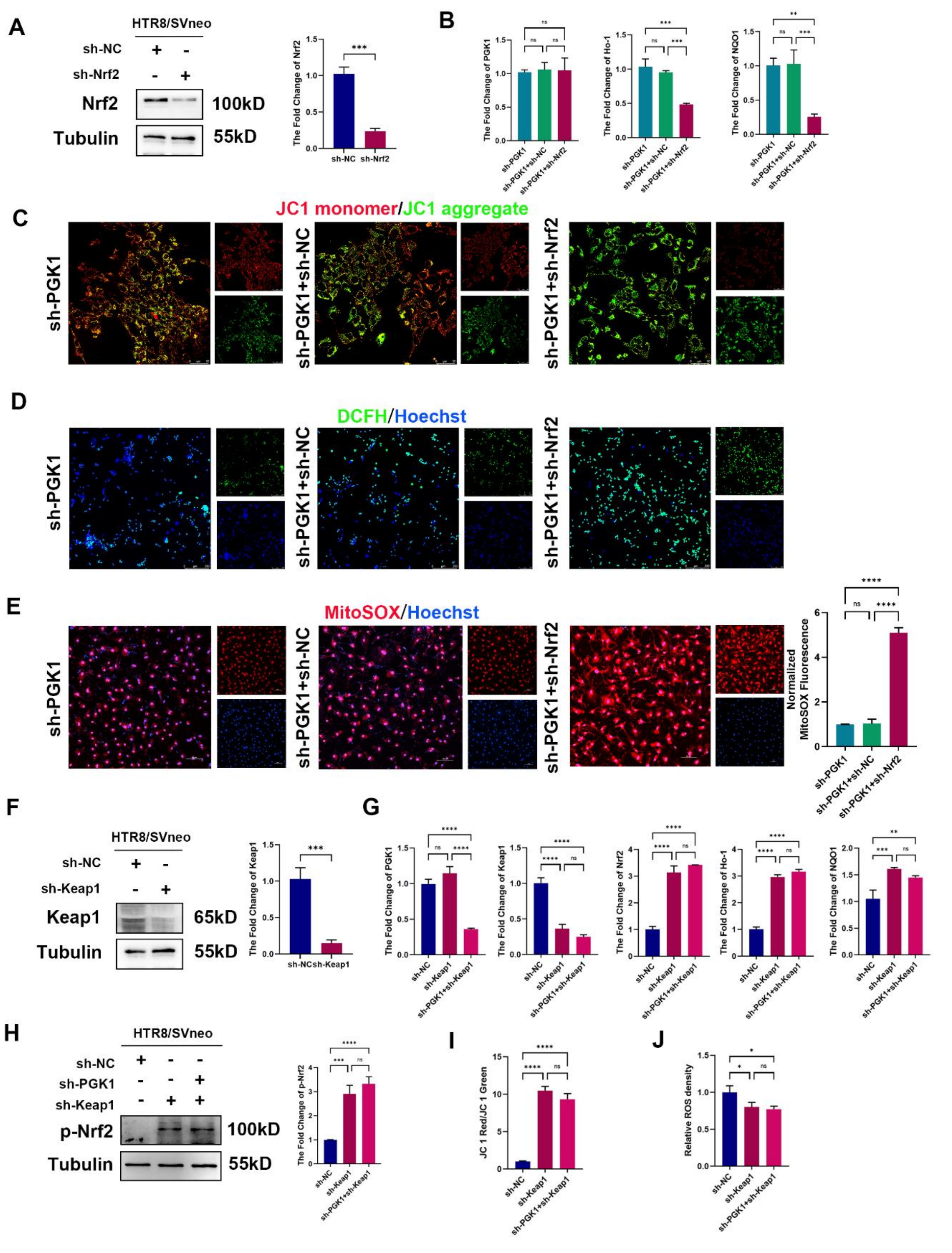

Supplementary Fig. 4 | Silencing PGK1 protects HTR8/SVneo cells by regulating Keap1-Nrf2 complex formation under HG conditions.

(A) The knockdown efficiency of Nrf2 was verified by Western blot; n = 3.

(B) Quantification of PGK1, Ho-1 and NQO1 in HTR8/SVneo cells stably transfected with non-targeting control shRNA, sh-PGK1+sh-NC, and sh-PGK1+sh-Nrf2; n = 3.

(C) Representative fluorescence images and quantification of MCMP in various groups; scale bar = 50 μm.

(D) Representative fluorescence images of ROS in various groups; scale bar = 100 μm.

(E) Representative fluorescence images and quantification of MitoSOX staining (red) in HTR8/SVneo cells, and Nuclei were counterstained with Hoechst (blue); scale bar = 50 μm; n = 3.

(F) The knockdown efficiency of Keap1 was verified by Western blot; n = 3.

(G) Quantification of PGK1, Keap1, Nrf2, Ho-1 and NQO1 in HTR8/SVneo cells stably transfected with sh-NC, sh-Keap1 or sh-Keap1+sh-PGK1; n = 3.

(H) Quantification of p-Nrf2 in HTR8/SVneo cells stably transfected with sh-NC, sh-Keap1, or sh-Keap1+sh-PGK1; n = 3.

(I) Quantification of MCMP in various groups; n = 3.

(J) Quantification of ROS in HTR8/SVneo cells; n = 3.

* represents $p < 0.05$, ** represents $p < 0.01$, *** represents $p < 0.001$, **** represents $p < 0.0001$ and ns represents $p > 0.05$.

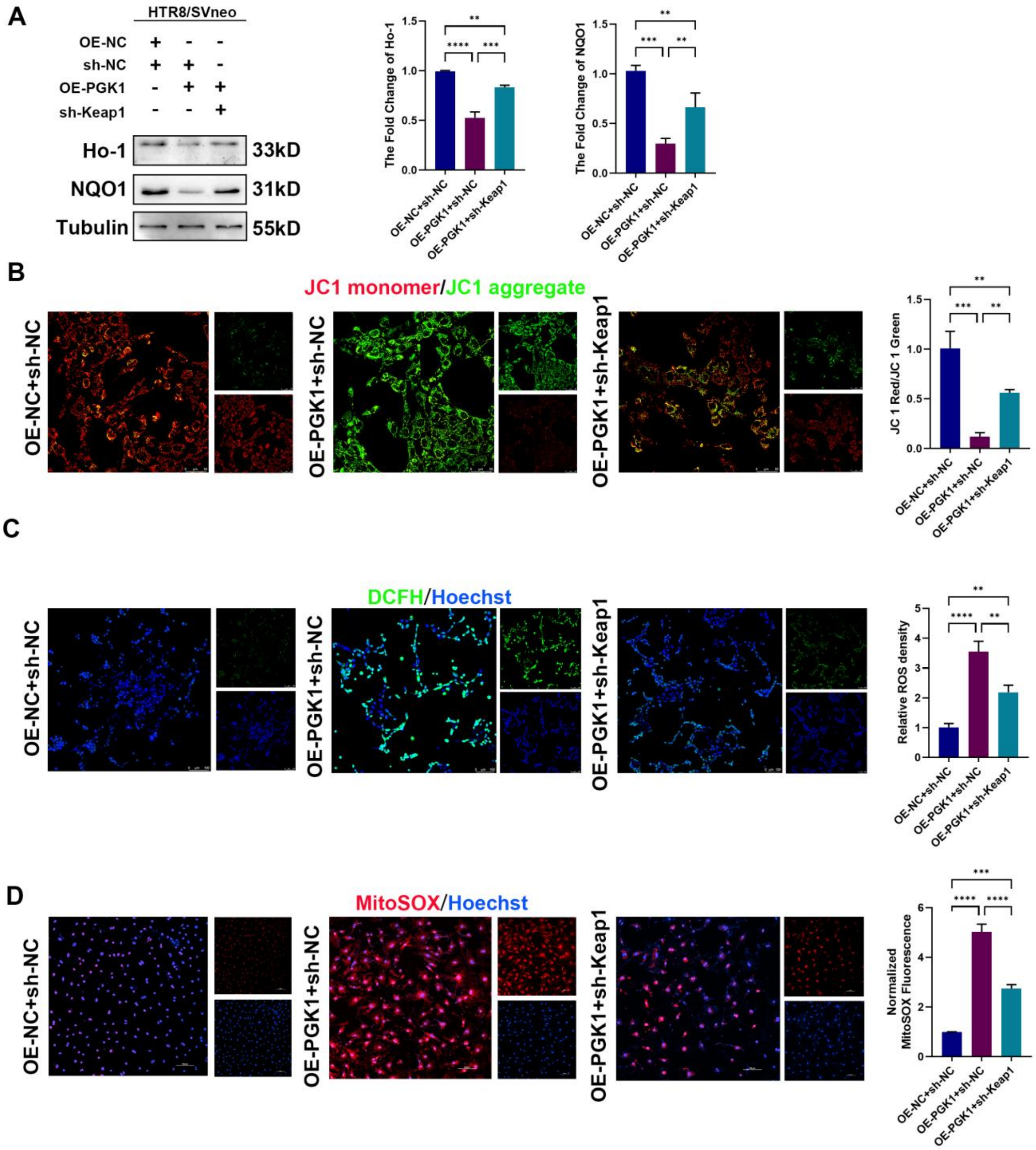

Supplementary Fig. 5 | Inhibition of Keap1 mitigates the adverse effects induced by PGK1.

(A) Representative Western blot images and quantification of Ho-1 and NQO1 in HTR8/SVneo cells stably transfected with a nonsense control for knockdown and overexpression (OE-NC+sh-NC); b. nonsense control for knockdown and PGK1 overexpression lentivirus (sh-NC+OE-PGK1); c. PGK1 overexpression lentivirus and Keap1 shRNA (OE-PGK1+sh-Keap1); n = 3.

(B) Representative fluorescence images and quantification of MCMP; scale bar = 50 μm; n = 3.

(C) Representative fluorescence images and quantification of ROS in various groups; scale bar = 250 μm; n = 3.

(D) Representative fluorescence images and quantification of MitoSOX staining (red) in HTR8/SVneo cells, and Nuclei were counterstained with Hoechst (blue); scale bar = 100 μm; n = 3.

* represents $p < 0.05$, ** represents $p < 0.01$, *** represents $p < 0.001$, **** represents $p < 0.0001$ and ns represents $p > 0.05$.

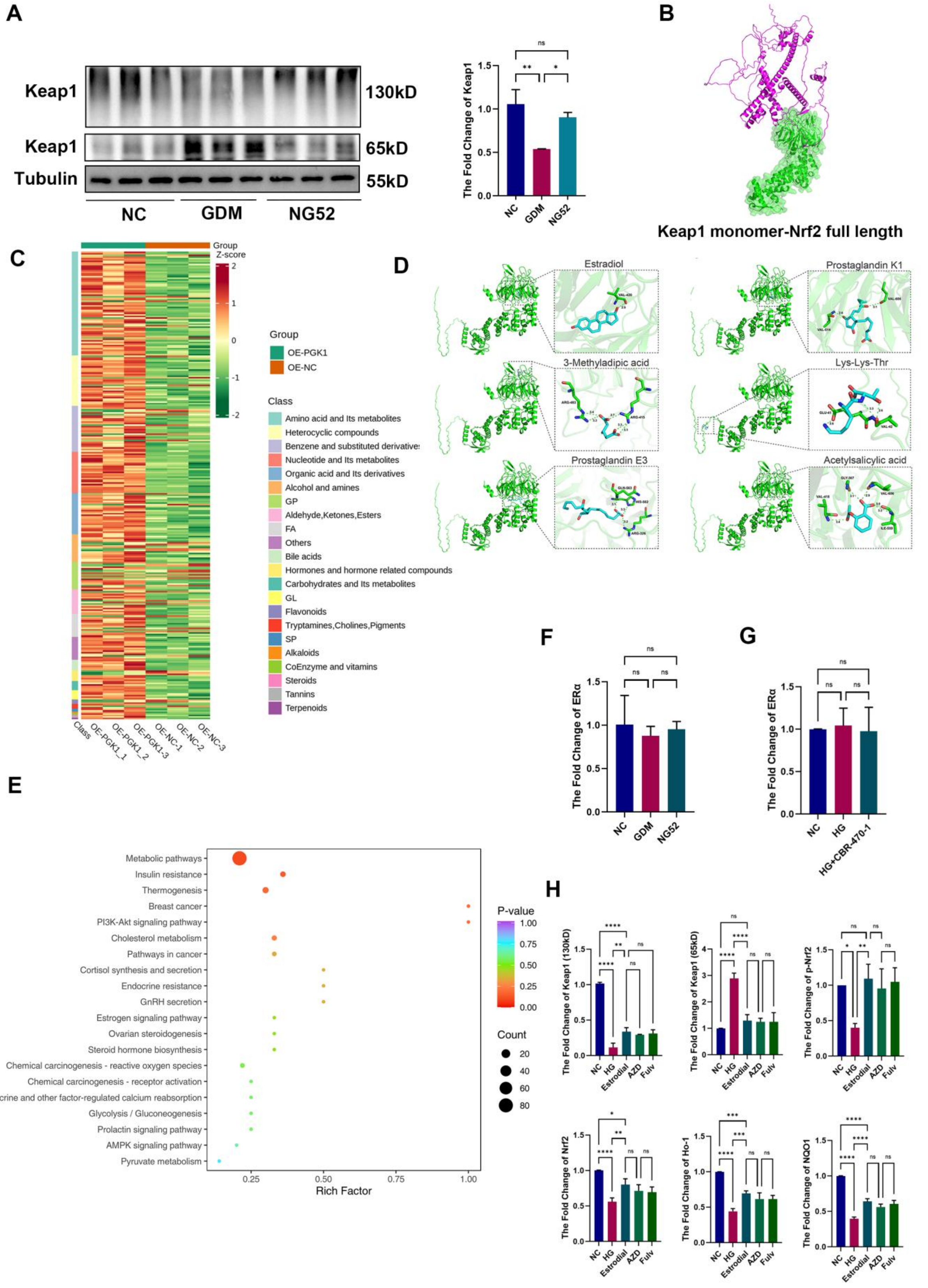

Supplementary Fig. 6 | Metabolomics of PGK1 overexpression.

(A) Representative Western blot images and quantification of Keap1 dimers in the control group, GDM group, and NG52 group; n = 3.
(B) The predicted binding complex models of monomeric Keap1 combined with full-length Nrf2.
(C) Heatmap of all metabolites with statistically significant differences.
(D) Molecular docking simulation of six metabolites screened with Keap1 monomer.
(E) Metabonomic pathway analysis was conducted using the KEGG database.
(F) Quantification of ERα in the three groups of mice; n = 3.
(G) Quantification of ERα in HTR8/SVneo cells; n = 3.
(H) Quantification of the levels of Nrf2, p-Nrf2, Ho-1, NQO1 and 130 kD, and 65 kD Keap1 in the HTR8/SVneo treated with estradiol combined with estrogen antagonist; n = 3.
* represents $p < 0.05$, ** represents $p < 0.01$ and ns represents $p > 0.05$.

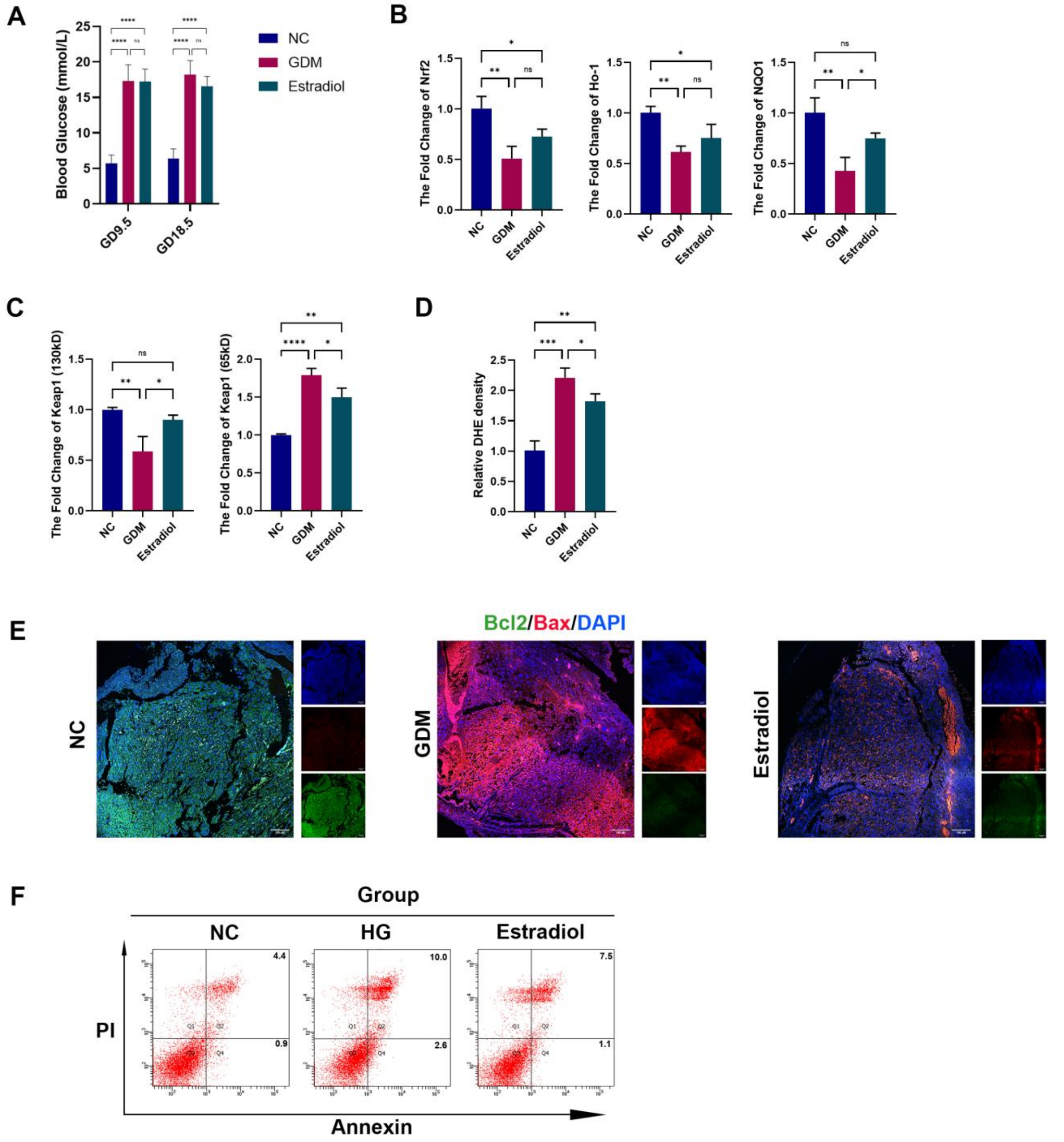

Supplementary Fig. 7 | Supplementation with estradiol partially mitigates HG-induced adverse pregnancy outcomes.

(A) Blood glucose of mice; n = 5.

(B) Quantification of the levels of Nrf2, Ho-1, and NQO1 in the NC group, GDM group, and Estradiol group; n = 3.

(C) Quantification of 130 kD and 65 kD Keap1 in the three groups; n = 3.

(D) Quantification of ROS levels in mouse placental tissues; n = 3.

(E) Bcl-2 protein levels (green), Bax protein levels (red) and DAPI coimmunofluorescence staining; scale bar = 100 μm.

(F) Apoptosis of HTR8/SVneo cells was tested via flow cytometry.

* represents $p < 0.05$, ** represents $p < 0.01$, *** represents $p < 0.001$, **** represents $p < 0.0001$ and ns represents $p > 0.05$.
